# Circulating Extracellular Vesicles Contain Liver-Derived RNA Species as Indicators of Severe Cholestasis-Induced Early Liver Fibrosis in Mice

**DOI:** 10.1089/ars.2021.0023

**Published:** 2022-03-17

**Authors:** Sharmila Fagoonee, Maddalena Arigoni, Marta Manco, Martina Olivero, Francesca Bizzaro, Cinzia Magagnotti, Annapaola Andolfo, Barbara Miniscalco, Marco Forni, Stefano Todeschi, Emanuela Tolosano, Elena Bocchietto, Raffaele Calogero, Fiorella Altruda

**Affiliations:** ^1^ Department of Biological Sciences, Institute of Biostructure and Bioimaging, National Research Council, Molecular Biotechnology Center, Turin, Italy.; ^2^ Department of Molecular Biotechnology and Health Sciences, Molecular Biotechnology Center, University of Turin, Turin, Italy.; ^3^ Department of Oncology, University of Turin, Torino, Italy.; ^4^ Abich S.r.l., Biological and Chemical Analysis, Verbania, Italy.; ^5^ ProMeFa, Proteomics and Metabolomics Facility, IRCCS, San Raffaele Scientific Institute, Milan, Italy.; ^6^ Department of Veterinary Sciences, University of Turin, Italy.

**Keywords:** bile duct ligation, cholestasis, circulating extracellular vesicles, biomarkers, liver fibrosis, mouse models

## Abstract

**Aims:**

Biliary diseases represent around 10% of all chronic liver diseases and affect both adults and children. Currently available biochemical tests detect cholestasis but not early liver fibrosis. Circulating extracellular vesicles (EVs) provide a noninvasive, real-time molecular snapshot of the injured organ. We thus aimed at searching for a panel of EV-based biomarkers for cholestasis-induced early liver fibrosis using mouse models.

**Results:**

Progressive and detectable histological evidence of collagen deposition and liver fibrosis was observed from day 8 after bile duct ligation (BDL) in mice. Whole transcriptome and small RNA sequencing analyses of circulating EVs revealed differentially enriched RNA species after BDL *versus* sham controls. Unsupervised hierarchical clustering identified a signature that allowed for discrimination between BDL and controls. In particular, 151 microRNAs (miRNAs) enriched in BDL-derived EVs were identified, of which 66 were conserved in humans. The liver was an important source of circulating EVs in BDL animals as evidenced by the enrichment of several hepatic mRNAs, such as *Albumin* and *Haptoglobin*. Interestingly, among experimentally validated miRNAs, miR192-5p, miR194-5p, miR22-3p, and miR29a-3p showed similar enrichment patterns also in EVs derived from 3,5-diethoxycarboncyl-1,4-dihydrocollidine-treated (drug-induced severe cholestasis) but not in mice with mild phenotype or non-cholestatic liver fibrosis.

**Innovation:**

A panel of mRNAs and miRNAs contained in circulating EVs, when combined, indicates hepatic damage and fibrosis in mice and represents promising biomarkers for human severe cholestasis-induced liver fibrosis.

**Conclusion:**

Analysis of EV-based miRNAs, in combination with hepatic injury RNA markers, can detect early cholestatic liver injury and fibrosis in mice. *Antioxid. Redox Signal.* 36, 480–504.

## Introduction

Cholestatic diseases, caused by impaired flow of bile from the liver to the duodenum, represent 10% of all liver diseases and impinge heavily on health care systems (7). Bile fluid comprises bile salts which are strong detergents and numerous endogenous products (*e.g.,* bilirubin) and potentially toxic compounds resulting from the clearance function of the liver (9). Hence, accumulation of bile compounds due to cholestasis causes unspecific cellular damage that starts a flow of inflammatory and fibrogenic events in the liver (42).

Cholangiocytes, hepatic stellate cells, and portal fibroblasts are the main players in the pathogenesis of cholestasis-induced fibrogenesis, with biliary obstruction leading to proliferation and activation of these cells (49). In the multifaceted and intertwined fibrogenetic process, any cell type involved can respond and release factors into the microenvironment and toward the bloodstream to exacerbate injury (25).

**Innovation**

Our data show that miR192-5p, miR194-5p, miR22-3p, and miR29a-3p were markedly increased in circulating EVs, in a reproducible way and specifically in severe cholestatic mouse models and correlated with the development of fibrosis. Thus, increase in the highly conserved EV-associated miR192-5p, miR194-5p, miR22-3p, and miR29a-3p (associated with severe cholestatic injury) and miR122-5p (a strong hepatocellular injury indicator), and in EV-based mRNAs, such as *Hp*, *TfR1*, and *Albumin* (good indicators of liver diseases), represent a promising panel of biomolecules to be further tested in humans as biomarkers for the early detection of cholestasis-induced liver fibrosis (Fig. 10).

If unchecked, fibrosis progresses to secondary biliary cholangitis and ultimately into liver failure and cancer (46). Multiple pathologies may represent the primary trigger of impaired bile flow, such as defects in key genes responsible for export of bile from hepatocytes to bile canaliculi (hepatocellular cholestasis), obstruction of bile ducts by gallstones or local tumor impingement (extrahepatic cholestasis), and drug-induced toxicity. Primary biliary cholangitis and primary sclerosing cholangitis are among the most common cholestatic liver diseases in the adult population, whereas biliary atresia and Alagille syndrome affect children (26, 52). One of the widely used experimental models to study cholestatic liver injury and fibrogenesis in rodents is the surgical bile duct ligation (BDL), which results in stereotypical histopathological alterations seen in human cholestasis (43).

The pathogenesis of cholestatic diseases remains understudied, despite the increase in incidence. Liver transplantation is the only definitive solution for end-stage patients (44). Early therapeutic intervention includes treatment with the choleretic, anti-cholestatic therapeutic bile acid ursodeoxycholic acid and with selective Farnesoid X receptor agonists such as obeticholic acid. Due to unresponsiveness to drugs or important side effects, development of other compounds is under way, which points out to the need for reliable biomarkers in the clinic (1). Monitoring of early fibrotic changes is tricky and usually not possible using conventional techniques such as transient elastography (commonly known as FibroScan) (32).

Liver biopsy (∼1/50,000th of the liver volume) followed by histological grading of fibrotic areas remains the gold standard. However, due to poor patient compliance, high risk, and heterogeneous deposit of collagen fibers in the injured liver, liver biopsy does not always allow early detection of fibrosis, thus soliciting the search for noninvasive alternatives (10). Biomarkers, such as circulating factors, provide a real-time molecular snapshot of the cells and tissues of origin. Currently available biomarkers for liver fibrosis detection have been reviewed elsewhere (33). However, search for new panels of biomolecules that can noninvasively and accurately detect early signs of liver fibrosis, when the latter is still reversible, is ongoing.

Within the past decade, extracellular vesicles (EVs) have emerged as critical mediators of intercellular communication. EVs transmit biomolecules, including nucleic acids, lipids, metabolites, and proteins, to regulate a plethora of biological processes (24). The term EV is used to describe a heterogeneous population of membrane-enclosed organelles formed by budding of the plasma membrane (microvesicles, 100–1000 nm in diameter) or released upon fusion of multivesicular bodies with the plasma membrane (exosomes, 40–100 nm in diameter) (6, 45).

Increasing evidence demonstrates that EV molecular payload representing the phenotypic status of the recipient cells is critical determinants of EV action on the evolution of fibrosis in several liver diseases (29). EV-mediated pathological alterations have been unraveled in several diseases, such as hepatocellular carcinoma or cholangiocarcinoma (5, 50). Moreover, EVs and their components, which reflect the dynamic status of underlying pathological conditions, may serve as new biomarkers for identifying and assessing molecular signatures associated with liver fibrosis.

**FIG. 1.**
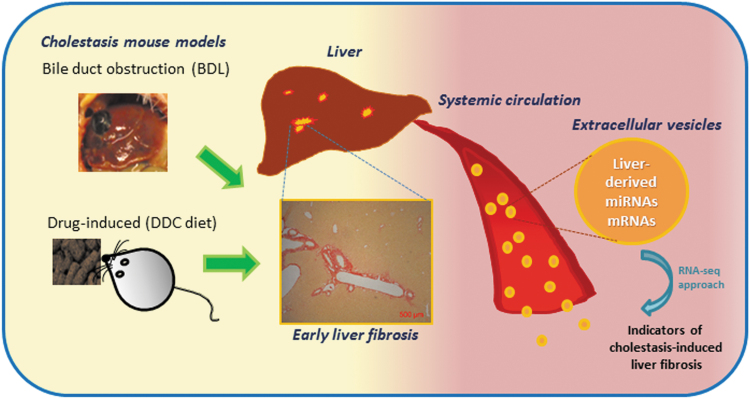
**Schematic representation of the potential of EV-based RNA species released from the liver following severe cholestatic injury for detection of early fibrosis.** BDL, bile duct ligation; DDC, 3,5-diethoxycarboncyl-1,4-dihydrocollidine; EV, extracellular vesicle. Color images are available online.

The EV contents are resistant to proteinase-dependent degradation and can hence be stably detected in the circulation, making these ideal biomarkers for a number of clinical applications (12). Thus, molecular characterization of circulating EV cargoes provides an unprecedented opportunity to detect the ongoing fibrogenesis, a common feature of most liver diseases, in a noninvasive and simple way at the preclinical level, which is a prelude to developing biomarkers of high utility in liver fibrosis early diagnosis and monitoring. The aim of the present study was to provide a high-throughput molecular characterization of the circulating EVs following cholestatic liver injury (obstruction, drug-induced, and genetic) in mice to identify biomolecules capable of indicating the surge of liver fibrosis (Fig. 1).

## Results

### Bile duct obstruction induced histopathologic and fibrotic changes in the liver

**FIG. 2.**
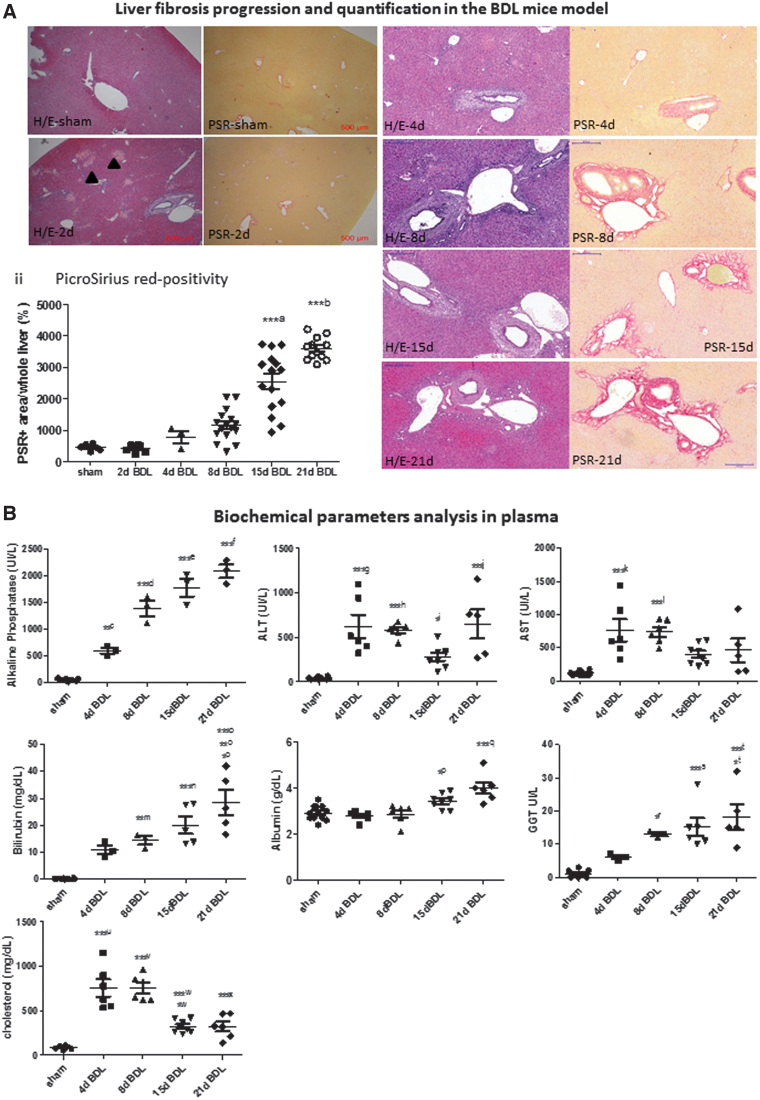

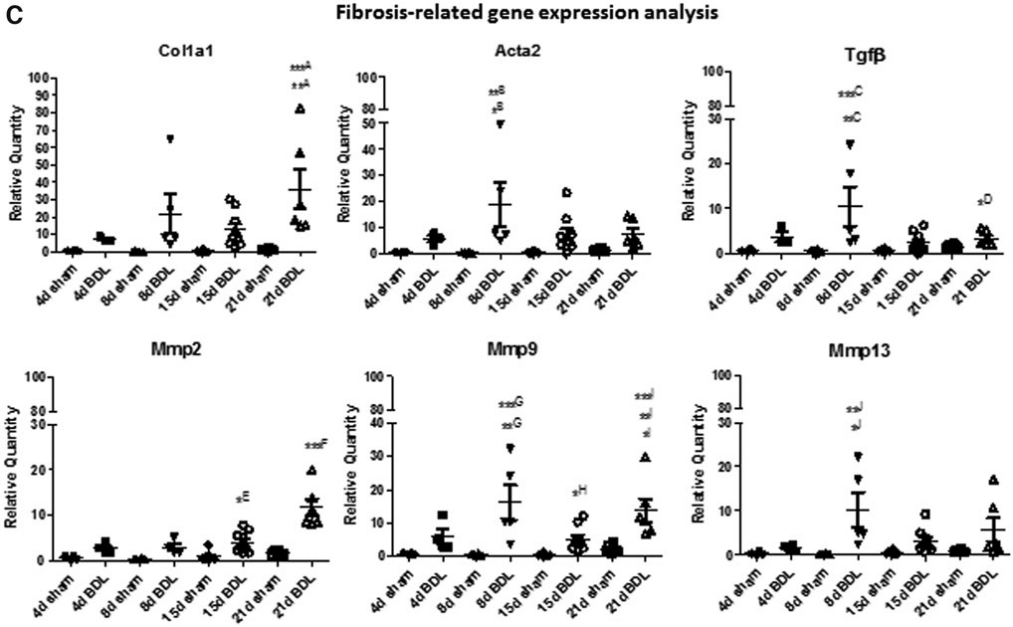
**Liver fibrosis in the BDL mice. (A)** (i) The typical appearance of liver tissue at representative time points after BDL using H/E staining and PSR staining are shown. Images were taken at 4 × magnification for sham and 2d BDL (scale bar: 500 μm) and at 10 × magnification (scale bar: 200 μm). Bile infacts areas (*arrowheads*) are seen as early as 2 days following BDL. (ii) PicroSirius red-positive areas were quantified using the ImageJ software (*n* ≥ 3); ***^a^15d BDL *versus* sham, 2d BDL, 4d BDL, 8d BDL, and 2d BDL, ***^b^21d BDL *versus* sham, 2d BDL, 4d BDL, 8d BDL, and 15d BDL. **(B)** Biochemical parameters analyzed in plasma at different time points after BDL *versus* sham-operated and healthy mice data are shown as the mean ± SEM (*n* ≥ 3); **^c^4d BDL *versus*4d sham, ***^d^8d BDL *versus* sham and 4d BDL, ***^e^15d BDL *versus* sham, 4d BDL, and 8d BDL, ***^e^15d BDL *versus* sham, 4d BDL, and 8d BDL, ***^f^21d BDL *versus* sham, 4d BDL, and 8d BDL, ***^g^4d BDL *versus* sham, ***^h^8d BDL *versus* sham, *^i^15d BDL *versus* 4d BDL, *^j^21d BDL *versus* 15d BDL, ***^j^15d BDL *versus* sham, ***^k^4d BDL *versus* sham, ***^l^8d BDL *versus* sham, **^m^8d BDL *versus* sham, ***^n^15d BDL *versus* sham, *^o^21d BDL *versus* 8d BDL, **^o^21d BDL *versus* 4d BDL, ***^o^21d BDL *versus* sham, *^p^15d BDL *versus* 4d BDL, ***^q^21d BDL *versus* sham, 4d BDL, and 8d BDL, *^r^8d BDL *versus* sham, ***^s^15d BDL *versus* sham, *^t^21d BDL *versus* 4d BDL, ***^t^21d BDL *versus* sham, ***^u^4d BDL *versus* sham, ***^v^8d BDL *versus* sham, *^w^15d BDL *versus* sham, ***^w^15d BDL *versus* 4d BDL and 8d BDL, ***^x^21d BDL *versus* 4d BDL and 8d BDL. **(C)** Expression of fibrogenesis-related genes *Col1A1*, *Acta2*, *Tgf-β*, *Mmp2*, *Mmp9*, and *Mmp2* was measured in mice livers by qRT-PCR. Data are shown as the mean ± SEM (*n* ≥ 3); **^A^21d BDL *versus* 4d sham, 21d sham, and 8d BDL, ***^A^21d BDL *versus* 15d sham, *^B^8d BDL *versus* 4d sham, ***^B^8d BDL *versus* 8d sham, 15d sham, and 21d sham, **^C^8d BDL *versus* 4d sham and 21d sham, ***^C^8d BDL *versus* 8d sham and 15d sham, *^D^21d BDL *versus* 8d BDL, *^E^15d BDL *versus* 8d sham, ***^F^21d BDL *versus* 4d sham, 8d sham, 15d sham, 21d sham, 4d BDL, 8d BDL and 21d BDL, **^G^8d BDL *versus* 4d sham and 21d sham, ***^G^8d BDL *versus* 8d sham and 15d sham, *^H^15d BDL *versus* 8d BDL, *I21d BDL *versus* 21d sham, **^I^21d BDL *versus* 4d sham and 8d sham, ***^I^21d BDL *versus* 15d sham, *^J^8d BDL *versus* 4d sham and 4d BDL, **^J^21d BDL *versus* 8d sham, 15d sham, and 21d sham. *p < 0.05, **p < 0.01, ***p < 0.001. H/E, hematoxylin and eosin; PSR, PicroSirius red; qRT-PCR, quantitative real-time polymerase chain reaction; SEM, standard error of the mean. Color images are available online.

To determine at which time point following BDL fibrosis is markedly detectable in mice, we performed a time course analysis of the livers. Histologically, Picrosirius red (PSR) staining evidenced periportal fibrosis as from day 8 post-BDL, and a progressive and statistically significant increase occurred up to 21 days (the last time point analyzed) concordant with previously reported data (Fig. 2A) (42). Thus, mice undergoing BDL for 8 and 15 days, presenting initial signs of fibrosis, were subsequently chosen for RNA sequencing (RNA-seq) analysis as described below. Cholestasis was confirmed by analysis of well-established clinical biochemical parameters (Fig. 2B).

A marked increase in hepatocellular (aspartate aminotransferase [AST], alanine aminotransferase [ALT]) and cholestatic injury markers (alkaline phosphatase [ALP], gamma glutamyl transferase [GGT], and bilirubin) was seen in the plasma of BDL mice *versus* sham and healthy controls. Moreover, total cholesterol as well as albumin showed a statistically significant increase after BDL (Fig. 2B). Serum cholinesterase levels did not decrease at 21 days after BDL indicating that liver function was not compromised (Supplementary Fig. S1).

As a result of bile duct obstruction, quantitative real-time polymerase chain reaction (qRT-PCR) analysis revealed that there was an increase in mRNA expression of transforming growth factor (*Tgf*)-*β*, the main profibrogenic molecule, of alpha-smooth muscle actin (*Acta2*), a marker of hepatic stellate cell activation, and of collagen fibrils (Collagen 1A1, *Col1a1*) as well as of matrix metalloproteinases (*mmp-2, -9,* and *-13*) compared with the respective sham controls (Fig. 2C). These results are in agreement with previously reported data (2). We further showed that liver injury and inflammation (interleukin-1β [*Il-1β*], tumor necrosis factor-α [*Tnfα*], C-X-C motif chemokine ligand 2 [*Cxcl2*]), detoxification/oxidative stress (glutathione S-transferase A2 [*Gsta2*]), and cholangiocyte proliferation (keratin 9, *Krt9*) were other major features involved (Supplementary Fig. S2).

### Characteristics of EVs enriched from plasma following BDL

**FIG. 3.**
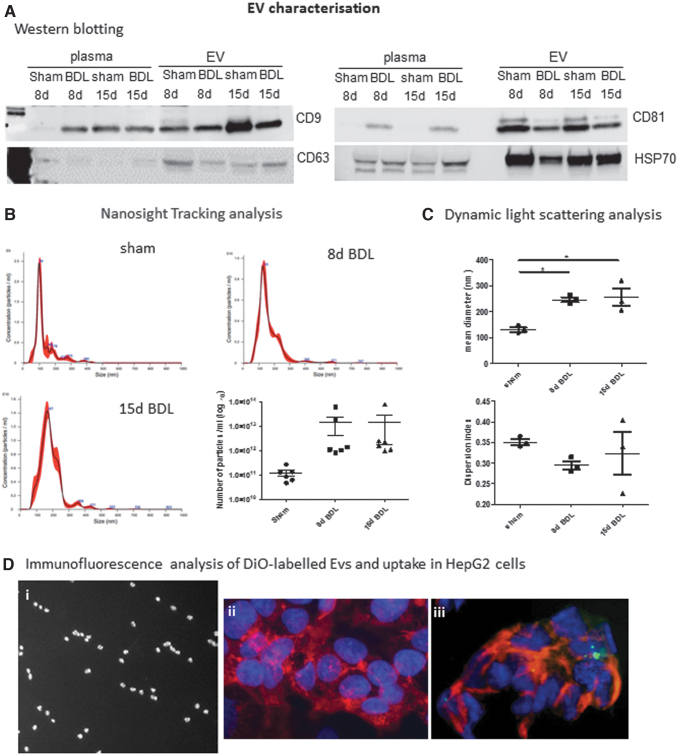
**Plasma EV characterization. (A)** The exosomal markers CD9, CD63, CD81, and HSP70 were analyzed by Western blot in whole plasma and EVs enriched from plasma at different time points after BDL and respective sham controls. Uncropped images of Western blots are shown in Figure S1. **(B)** Size and concentration of EVs were analyzed by NTA. Representative images are shown. Mean ± SEM of number of particles per milliliter EV was calculated at each time point (*n* = 6). **(C)**. DLS analysis shows mean diameter and dispersion index of EVs after BDL (*n* = 3). **(D)** (i) Vybrant DiO-stained EVs visualized under fluorescence microscope, at 100 × magnification; (ii) Serum or (iii) labeled EVs (*green*) were added to HepG2 cells for 24 h and then stained with phalloidin (*red*) and DAPI (*blue*) for imaging at 40 × magnification on a fluorescence microscope. *p < 0.05. DAPI, 4′,6-diamidino-2-phenylindole; DiO, DiOC18(3) (3,3′-dioctadecyloxacarbocyanine perchlorate); DLS, dynamic light scattering; NTA, nanoparticle tracking analysis. Color images are available online.

Cargoes of EVs isolated from plasma and serum have been proposed as biomarkers for several diseases, including alcohol- and drug-induced liver injury (15, 37). We investigated herein whether EVs released in the circulation upon cholestatic liver injury in mice could serve as prognostic and diagnostic biomarkers for early fibrosis detection. Total circulating EVs were enriched from plasma of BDL mice at days 8 and 15, when early signs of fibrosis were detected, and analyzed for the presence of exosomal markers (CD9, CD63, CD81, HSP70) (Fig. 3A). Nanoparticle tracking analysis (NTA) revealed a peak size of EVs of ∼100 nm in sham controls and ∼200 nm in BDL mice.

As expected, there was a rise, although not statistically significant, in EV concentration in the plasma of BDL mice compared with sham controls (Fig. 3B). Dynamic light scattering (DLS) analyses confirmed a statistically significant increase in size of EVs following BDL with respect to controls (Fig. 3C). No statistically significant difference in dispersion index (heterogeneity) of EVs was seen among experimental groups. EVs were also labeled for visualization and uptake in HepG2 cells. As shown in Figure 3D, DiO-labeled EVs were efficiently taken up by HepG2 cells.

### EVs enriched from plasma of BDL mice were loaded with liver-derived RNA species

To identify circulating EV-associated RNA species differentially enriched upon fibrosis development in the cholestatic liver, RNA-seq analysis of small noncoding RNAs as well as of whole transcriptome was performed on RNA extracted from EVs (Supplementary Fig. S3). EVs from plasma of mice undergoing BDL for 8 and 15 days were compared with their respective sham-operated controls.

**FIG. 4.**
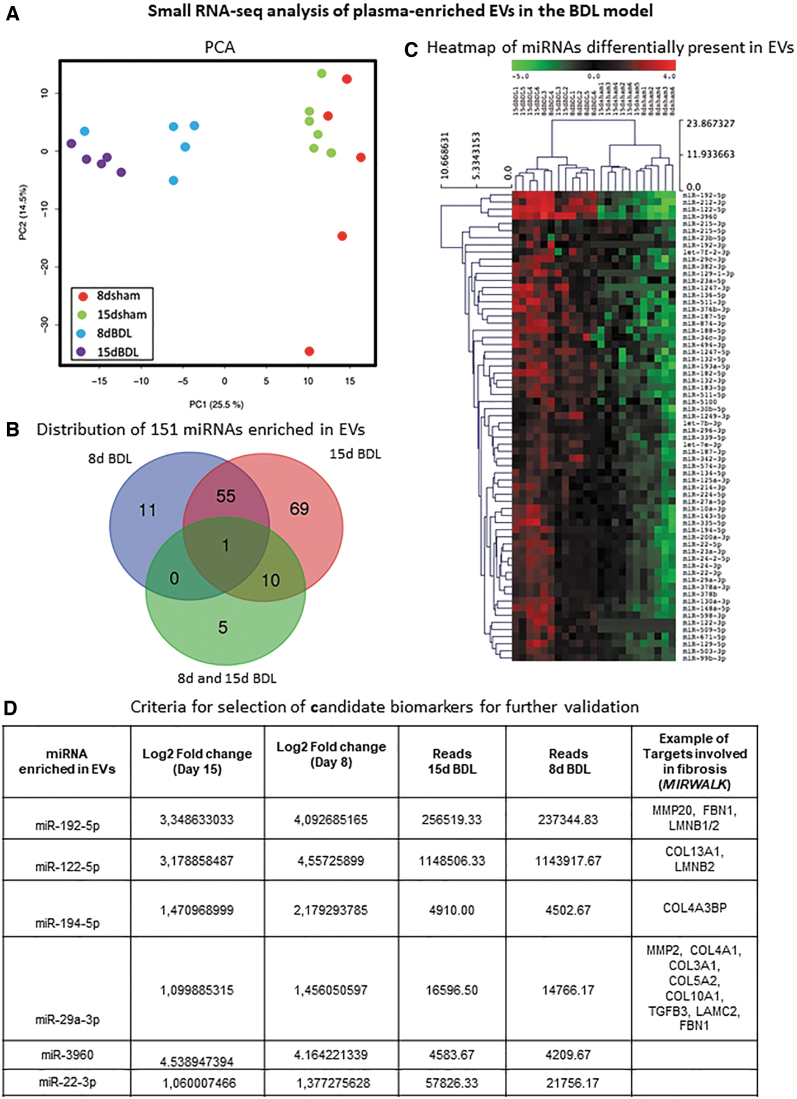
**Small RNA-seq analysis of circulating EVs in BDL mice. (A)** PCA analysis. **(B)** Venn diagram showing the distribution of murine miRNAs enriched in plasma-derived EVs after BDL; miRNAs upregulated at 8d BDL only or 15d BDL only or progressively from 8d to 15d are represented. **(C)** Heat map showing 66 conserved miRNAs enriched in EVs after BDL with respect to sham controls. **(D)** Criteria used to select miRNAs for validation are shown. PCA, principal component analysis. Color images are available online.

Regarding small RNA-seq analysis, an average of 1 million reads per sample was obtained. Principal component analysis (PCA) revealed that data sets of all BDL mice grouped together and did not overlap with sham controls (Fig. 4A). We identified 250 differentially expressed EV-associated microRNAs (miRNAs). In view of a possible translation of these candidate biomarkers onto human cholestasis-induced liver fibrosis, we focused our work only on upregulated miRNAs (*p* value ≤0.01 and an absolute value of the log2 ratio ≥1). One hundred fifty-one miRNAs were detected in EVs as differentially present between BDL and sham controls (Fig. 4B). Among these 151 miRNAs upregulated following BDL, 66 were found conserved in humans (Fig. 4C and Supplementary Table S3).

**FIG. 5.**
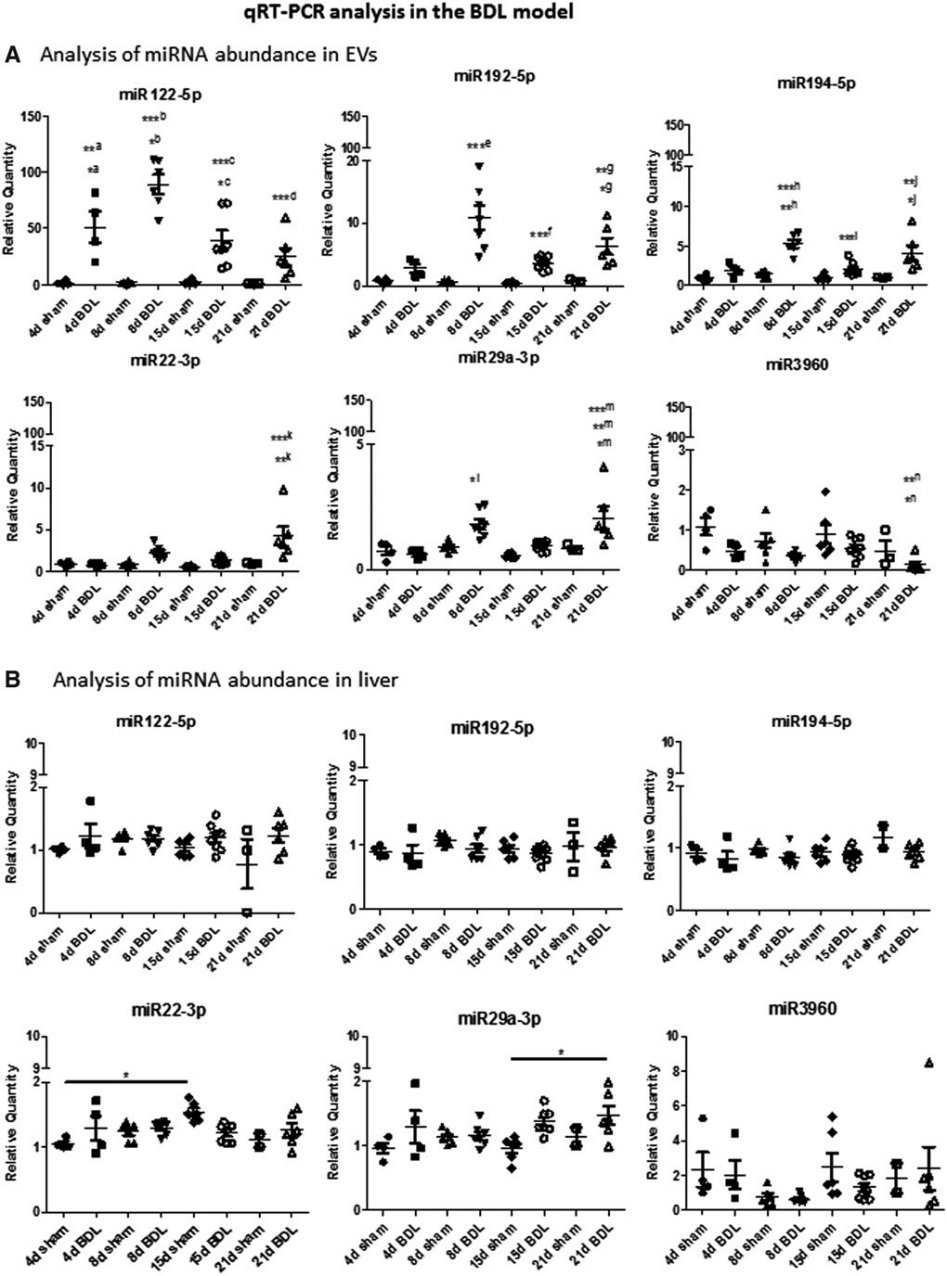
**Analysis of miRNAs by qRT-PCR. (A)** Differential expression of six miRNAs chosen from the RNA-seq experiment was confirmed by qRT-PCR. Data show mean ± SEM of abundance of each miRNA in circulating EVs obtained from mice undergoing BDL (different time points) and respective sham controls (*n* ≥ 4); *^a^4d BDL *versus* 21d sham, **^a^4d BDL *versus* 4d sham, 8d sham, and 15d sham, *^b^8d BDL *versus* 4d BDL, ***^b^8d BDL *versus* 4d sham, 8d sham, 15d sham, and 21d sham, *^c^15d BDL *versus* 4d sham, 8d sham, and 15d sham, ***^c^15d BDL *versus* 8d BDL, ***^d^21d BDL *versus* 8d BDL, ***^e^8d BDL *versus* 4d sham, 8d sham, 15d sham, 21d sham, and 8d BDL, ***^f^15d BDL *versus* 8d BDL, *^g^21d BDL *versus* 4d sham and 8d sham, **^g^21d BDL *versus* 15d sham, **^h^8d BDL *versus* 4d BDL, ***^h^8d BDL *versus* 4d sham, 8d sham, 15d sham, and 21d sham, ***^i^15d BDL *versus* 8d BDL, *^j^21d BDL *versus* 15d BDL, **^j^21d BDL *versus* 4d sham, 8d sham, 15d sham, and 21d sham, **^k^21d BDL *versus* 4d sham, 21d sham, 4d BDL, and 15d BDL, ***^k^21d BDL *versus* 8d sham and 15d sham, *^l^8d BDL *versus* 15d sham and 4d BDL, *^m^21d BDL *versus* 21d sham, **^m^21d BDL *versus* 4d sham, ***^m^21d BDL *versus* 8d sham, 15d sham, 4d BDL, and 15d BDL, *^n^21d BDL *versus* 15d sham, **^n^21d BDL *versus* 4d sham. **(B)** Data show mean ± SEM of abundance of each miRNA in the liver of mice undergoing BDL and respective sham controls (*n* ≥ 3). *p < 0.05, **p < 0.01, ***p < 0.001.

miRNAs potentially involved in cholestasis-induced liver fibrosis were selected according to the following criteria for further confirmation by a second RNA-seq and qRT-PCR: (i) increase in abundance in circulating EVs, (ii) log2-fold change ≥2, (iii) reads ≥1000 in at least one of the conditions, and (iv) putative miRNA target genes, identified using the Mirwalk 2.0 software (Fig. 4D), must be involved in fibrosis. Data validation was performed by sequencing of circulating EV RNAs obtained from day 8 BDL and sham-operated mice, as shown in Supplementary Figure S4. Abundance of selected miRNAs (miR122-5p, miR194-5p, miR192-5p, miR22-3p, miR29a-3p, and miR-3960) in EVs was further confirmed at day 8 and 15 post-BDL compared with respective sham controls using qRT-PCR (Fig. 5 and Supplementary Fig. S4).

A prior time point, day 4, was included in this analysis to investigate whether the EV-associated miRNAs were detected earlier than the histological fibrotic changes at day 8 (Fig. 2A). Moreover, the trend in abundance of selected miRNAs was also assessed at day 21 post-BDL, a time when slightly advanced fibrosis with respect to day 15 was observed. In particular, by using qRT-PCR, a statistically significant increase in miR122-5p as from day 4 after BDL was observed, whereas miR192-5p and miR194-5p showed a statistically significant increase after 8 days post-BDL, with respect to sham controls (Fig. 5A).

MiR122-5p and miR192-5p, known hepatotoxicity biomarkers, were the most represented miRNAs following BDL (Supplementary Fig. S4). Interestingly, the fibrosis-associated miR29a-3p, as well as the exosome-associated miR22-3p, showed a statistically significant increase at day 8 and 21, and day 21, respectively, after BDL.

To investigate whether the circulating EV RNA contents and abundance reflect that of the tissue of origin, we extracted RNA from the liver of mice from which EVs were prepared. qRT-PCR analysis revealed that, in the liver, there was a statistically significant increase only in miR29a-3p at day 21 after BDL. The other miRNAs did not show marked changes in expression in the liver upon BDL (Fig. 5B). Overall, no great difference in the abundance of the EV-associated miRNAs was observed in BDL with respect to sham control livers, and EV miRNA content did not correlate with liver miRNA content.

**FIG. 6.**
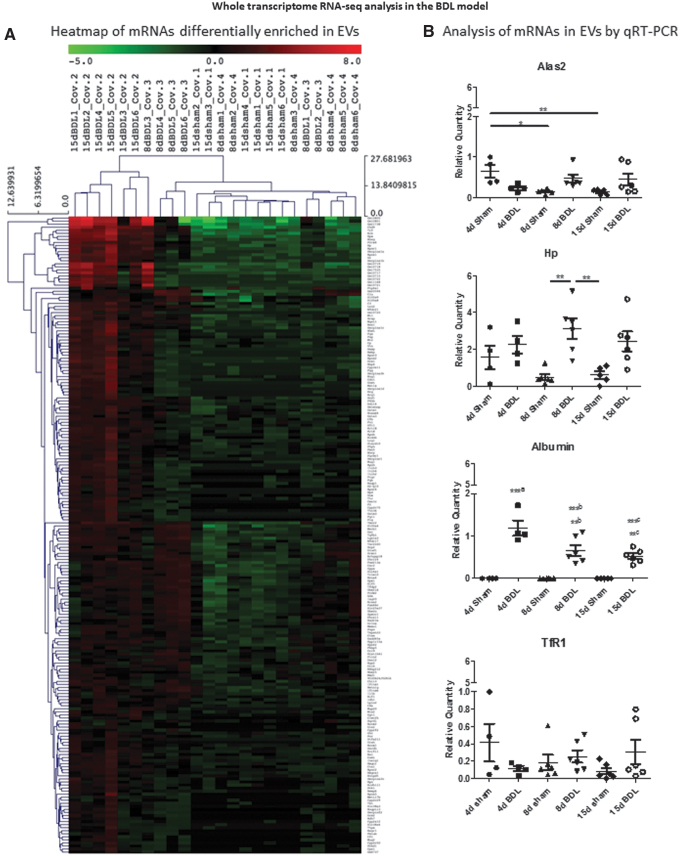
**Whole transcriptome analysis of circulating EVs in BDL mice. (A)** Heat map showing 209 mRNAs enriched in EVs after BDL with respect to sham controls. **(B)** Differential expression of selected mRNAs was confirmed by qRT-PCR. Data show mean ± SEM of abundance of each mRNA in the circulating EVs of mice undergoing BDL and respective sham controls (*n* ≥ 4); ***^a^4d BDL *versus* 4d sham, 8d sham, and 15d sham, **^b^8d BDL *versus* 4d BDL, ***^b^8d BDL *versus* 4d sham, 8d sham, and 15d sham, **^c^15d BDL *versus* 4d sham, 8d sham, and 15d sham, ***^c^21d BDL *versus* 4d BDL. *p < 0.05, **p < 0.01, ***p < 0.001. Color images are available online.

We also analyzed the whole set of RNA species present in the circulating EVs after BDL. This analysis pointed to the protein-coding RNAs as the most abundant RNA species found in the plasma-derived EVs (Supplementary Fig. S5 and Supplementary Table S4). Small non-coding RNAs and long non-coding RNAs were also differentially present among the different time points (Supplementary Fig. S5). Two hundred nine transcripts were more abundant in the BDL groups with respect to sham controls (Fig. 6A and Supplementary Table S5). Gene set enrichment (Enrich R, https://maayanlab.cloud/Enrichr) analysis (https://pubmed.ncbi.nlm.nih.gov/27141961) showed that the mRNAs enriched in EVs following BDL were liver specific (Supplementary Fig. S6).

Of the 209 transcripts, those also known to be abundantly expressed in the liver were chosen for further validation by qRT-PCR analysis (Fig. 6B). Haptoglobin (HP), an acute phase hepatic protein involved in hemoglobin metabolism, statistically significantly increased in circulating EVs at day 8 post-BDL compared with sham controls (19). The mRNA of *Albumin*, the most abundant circulating protein, was present in EVs only after BDL (as early as day 4), whereas the abundance of 5-aminolevulinate synthase 2 gene (*Alas2*), which encodes the erythroid-specific isoform of the enzyme that initiates heme biosynthesis, and of transferrin receptor 1 (*TfR1*) mRNAs in EVs showed no pronounced changes upon cholestatic injury.

**FIG. 7.**
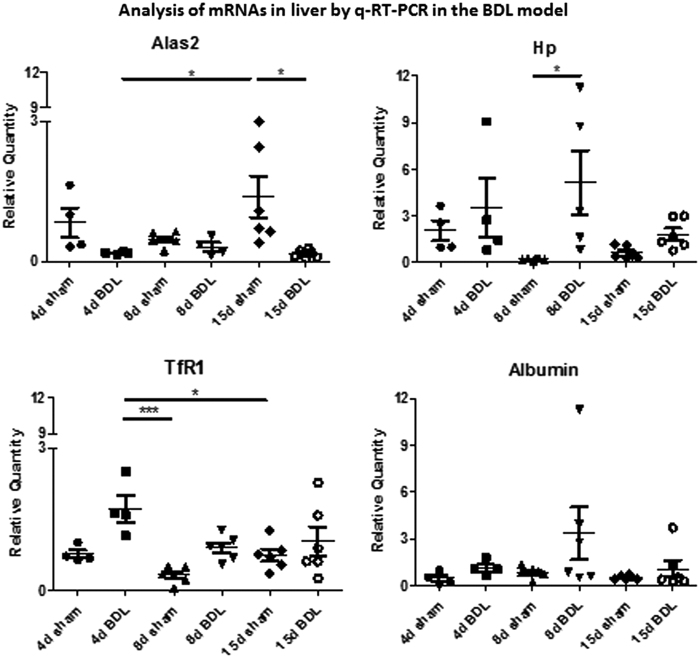
**Analysis of mRNAs in the liver by qRT-PCR.** Data show mean ± SEM of expression of each mRNA in the liver of mice undergoing BDL (different time points) and respective sham controls (*n* ≥ 4). *p < 0.05, ***p < 0.001.

Further analysis, by qRT-PCR, of the expression of these mRNAs in the BDL mice livers showed that *Alas2* showed a statistically significant decrease at days 4 and 15, *Hp* and *TfR1* increased statistically significantly at days 8 and 4, respectively, whereas *Albumin* remained unchanged compared with those of respective sham controls (Fig. 7). The expression of *Albumin* in the liver once again confirmed that liver function was not affected by BDL at early time points. Thus, our data show that the abundance of mRNAs, such as of *Hp* and *Albumin*, in circulating EVs can be also used as potential biomarkers of cholestatic liver.

### EV enrichment of RNA molecules similar to BDL mice is observed upon severe cholestasis-induced fibrosis

**FIG. 8.**
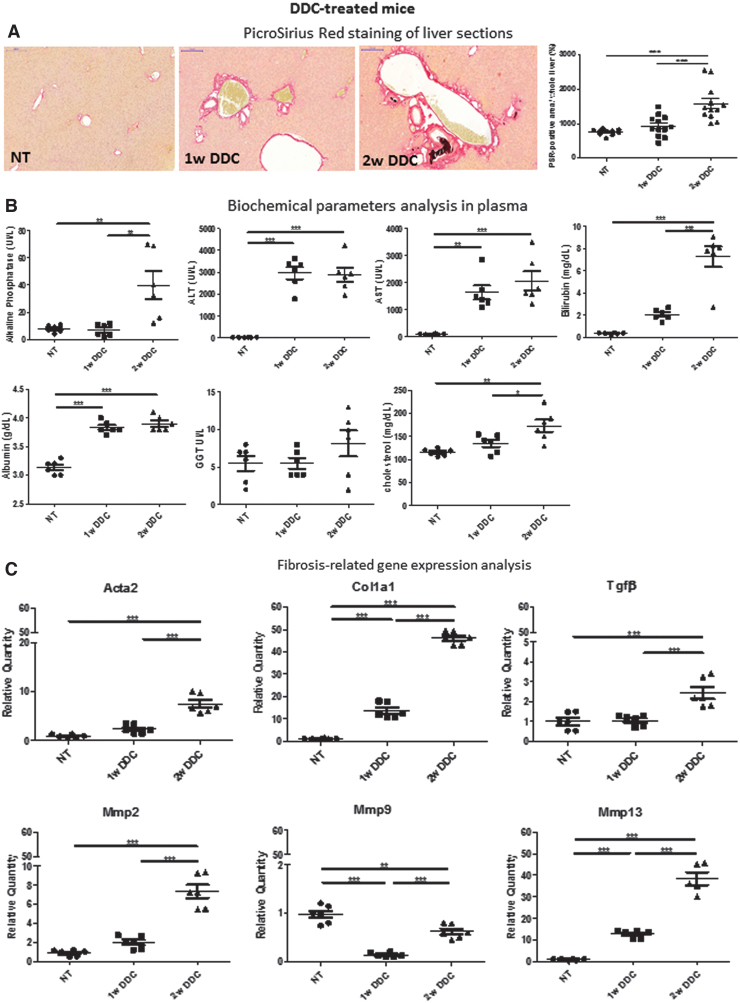

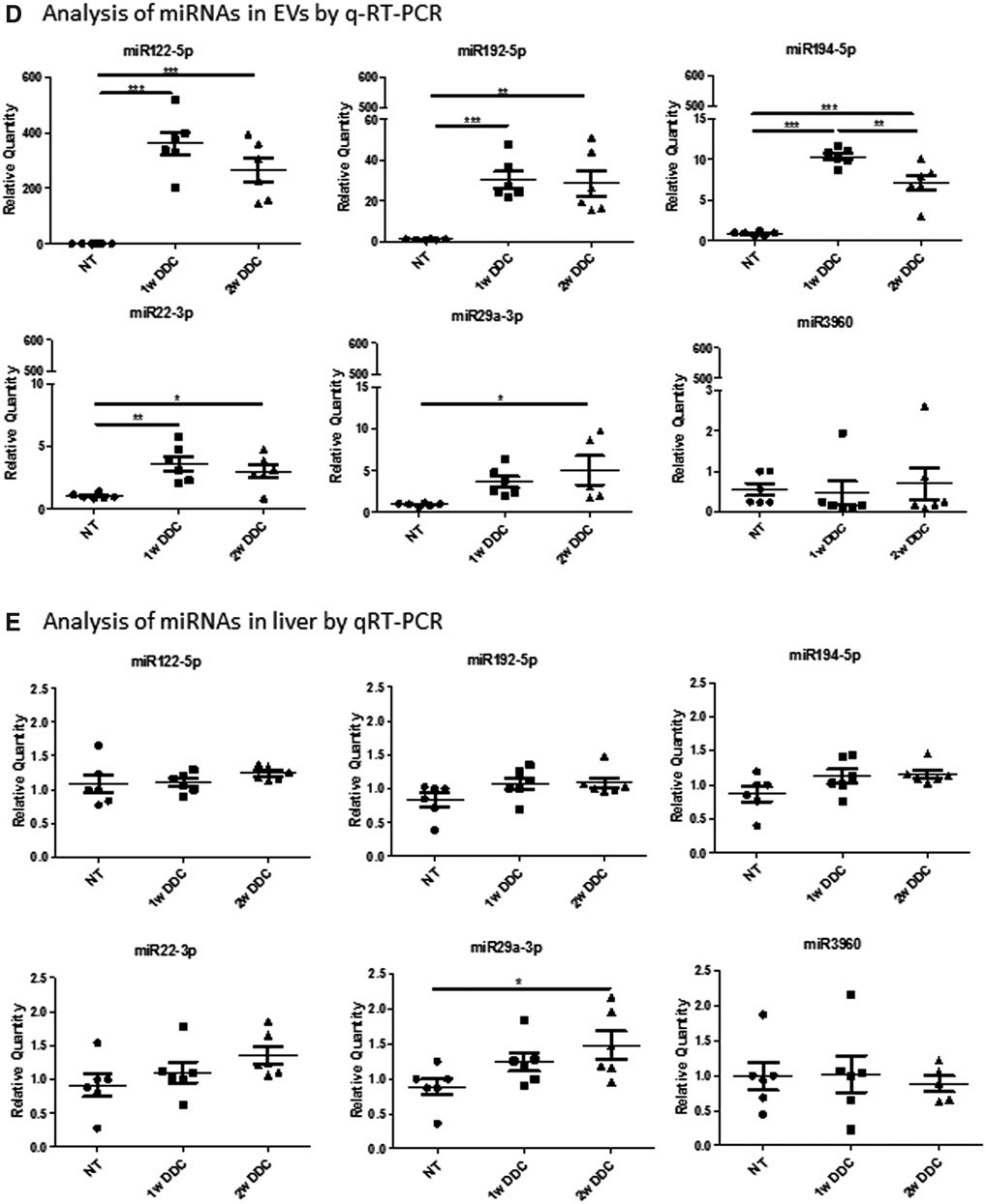

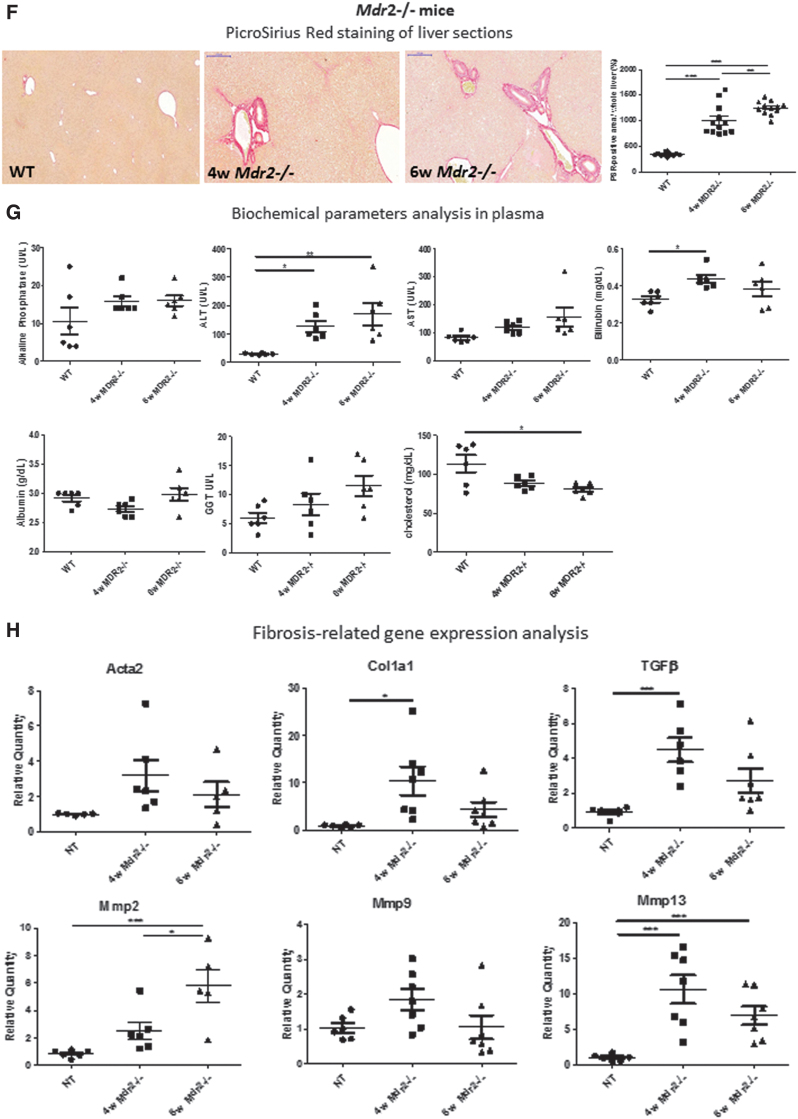

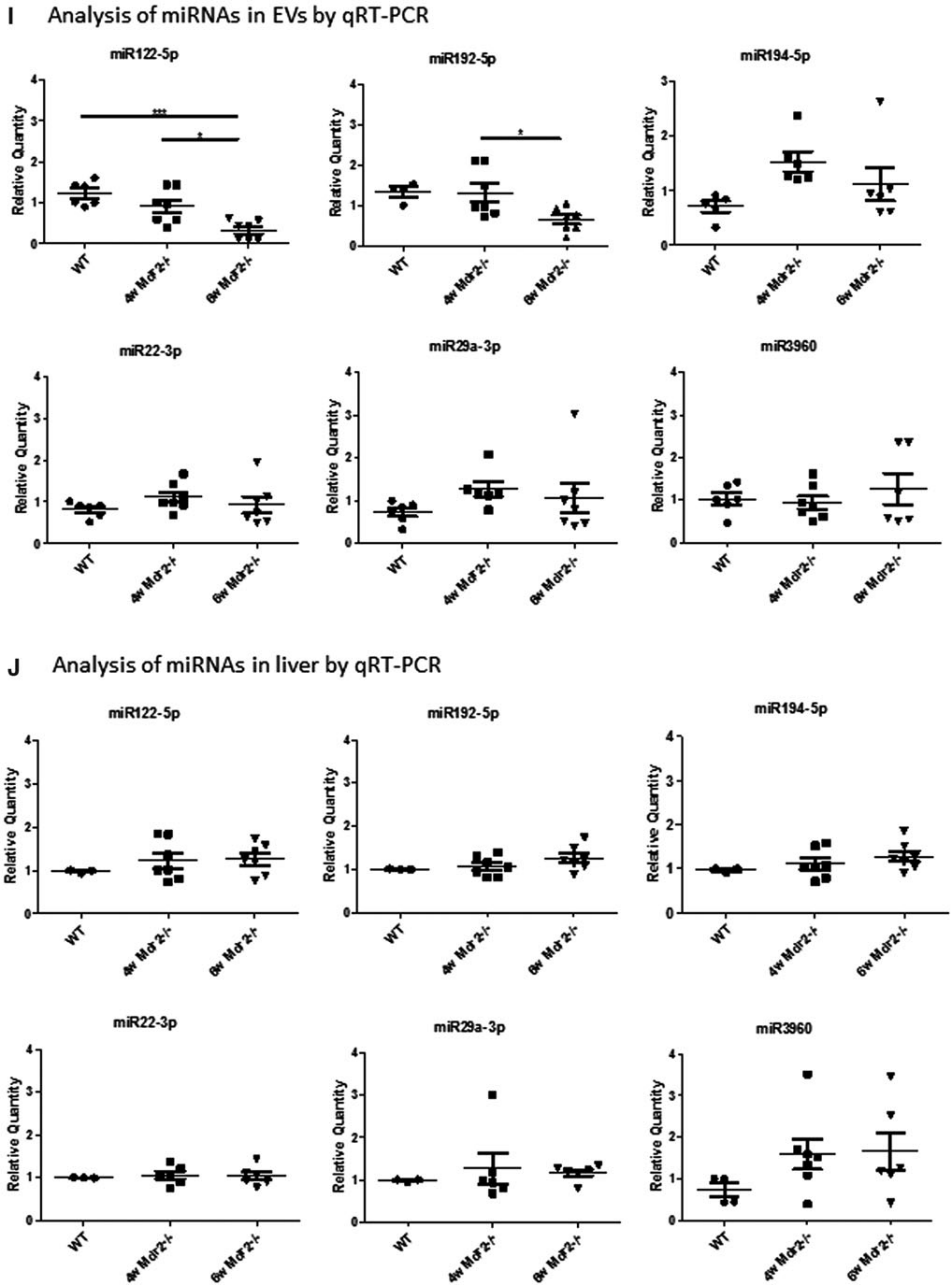

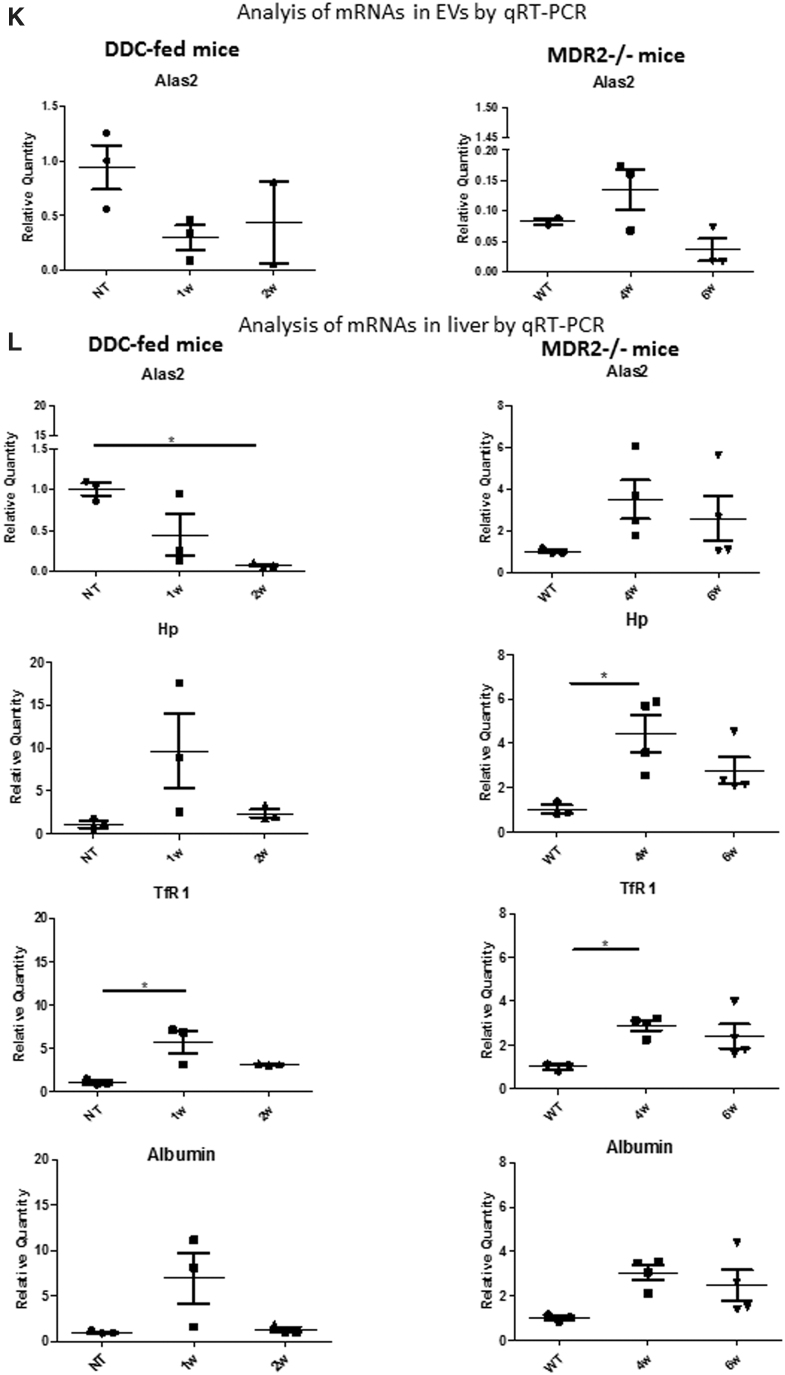
**Analysis of DDC-fed and** *Mdr*2-/- **cholestatic mice. (A)** Collagen deposits (*red*) were analyzed by PSR staining in the livers of mice fed on DDC-diet for 1 and 2 weeks with respect to NT. **(B)** Biochemical parameters analysis in DDC-treated mice. **(C)** Fibrosis-related gene expression analysis DDC-treated mice (*n* = 6). **(D)** miRNAs identified as enriched in circulating by RNA-seq in the BDL model were analyzed by qRT-PCR in the DDC-fed mice. Data show mean ± SEM of abundance of each miRNA in circulating EVs (*n* = 6). **(E)** Data show mean ± SEM of abundance of each miRNA in the liver of DDC-fed mice by qRT-PCR (*n* = 6). *p < 0.05, **p < 0.01, ***p < 0.001. **(F)** Collagen deposits (*red*) were analyzed by PSR staining in the *Mdr2-/-* mice livers at 4 and 6 weeks of age with respect to WT controls (*n* = 12). **(G)** Biochemical parameters analysis in *Mdr2-/-* mice (*n* = 6). **(H)** Fibrosis-related gene expression analysis *Mdr2-/-* mice. **(I)** miRNAs identified as enriched in circulating by RNA-seq in the BDL model were analyzed by qRT-PCR in the *Mdr2-/-* mice (*n* ≥ 5). Data show mean ± SEM of abundance of each miRNA in circulating EVs (*n* ≥ 4). **(J)** Data show mean ± SEM of abundance of each miRNA in the liver of *Mdr*2-/- mice by qRT-PCR (*n* ≥ 3). *p < 0.05, **p < 0.01, ***p < 0.001. **(K)** mRNAs identified as enriched in circulating by RNA-seq in the BDL model were analyzed by qRT-PCR in the DDC-fed mice and *Mdr*2-/- mice, respectively. Data show mean ± SEM of abundance of Alas2 in circulating EVs (*n* = 3). **(L)** The expression of the mRNAs in the DDC-treated and *Mdr2*-/- mouse livers was also analyzed, and data show mean ± SEM (*n* ≥ 3). *p < 0.05, **p < 0.01, ***p < 0.001. NT, non-treated mice; WT, wild type. Color images are available online.

Data obtained from the BDL model were further assessed, by qRT-PCR, in mice with a genetic defect in the hepatocyte apical canaliculi, MDR2, and in mice with drug-induced cholestasis. The latter was achieved by supplementing the diet with 3,5-diethoxycarboncyl-1,4-dihydrocollidine (DDC). DDC-treated mice livers showed the typical onion skin type-like periductal fibrosis as from 1 week of treatment (Fig. 8A), whereas *Mdr2-/-* mice showed mild cholestasis and mild age-dependent increase in periductular extracellular matrix deposition, as previously reported (Fig. 8F) (20, 36). Biochemical parameters assessment and analysis of liver gene expression confirmed these histological changes in the DDC-treated and *Mdr2*-/- mice (Fig. 8B, C, G, and H, respectively, and Supplementary Fig. S2).

Regarding the abundance of miRNAs, miR122-5p, miR194-5p, miR192-5p, miR22-3p, and miR29a-3p increased statistically significantly in circulating EVs of DDC-treated mice with respect to standard chow-fed controls (non-treated mice), whereas miR-3960 level did not change markedly (Fig. 8D). In *Mdr2-/-* mice, a statistically significant decrease in miR122-5p and miR192-5p was observed in EVs at 6 weeks of age with respect to wild-type (WT) controls (Fig. 8I). The other miRNAs under study did not show statistically significant changes in *Mdr2-/-* mice EVs compared with WT ones. Thus, the six miRNAs found enriched in EVs after BDL were found similarly abundant in EVs from severe DDC-induced cholestatic mice (Figs. 5 and 8D).

We further analyzed the expression of these miRNAs in the liver of DDC-treated and *Mdr2-/-* mice (Fig. 8E, J, respectively). Interestingly, only miR29a-3p was statistically significantly overexpressed in the mouse liver after 2 weeks of DDC treatment, whereas in the *Mdr2-/-* mice liver, no statistically significant changes in the expression of these miRNAs were observed compared with WT controls.

Regarding the expression of EV-associated mRNAs revealed by whole transcriptome analysis in the BDL model, we could detect only *Alas2* mRNA in the circulating EVs of DDC-treated and *Mdr2-/-* mice, but there was no pronounced difference in its abundance between cholestatic condition and controls (Fig. 8K). However, in the liver of DDC-fed mice, there was a statistically significant decrease in *Alas2* expression after 2 weeks of treatment, whereas 1 week DDC-fed and 4 weeks old *Mdr2-/-* mice showed statistically significantly increase in *TfR1* expression and *TfR1* and *Hp* expression, with respect to nontreated mice and WT controls, respectively (Fig. 8L). *Albumin* expression did not change significantly in the livers of these mice.

### Selected liver-derived RNA species abundance in EVs does not vary significantly upon carbon tetrachloride-induced liver injury

**FIG. 9.**
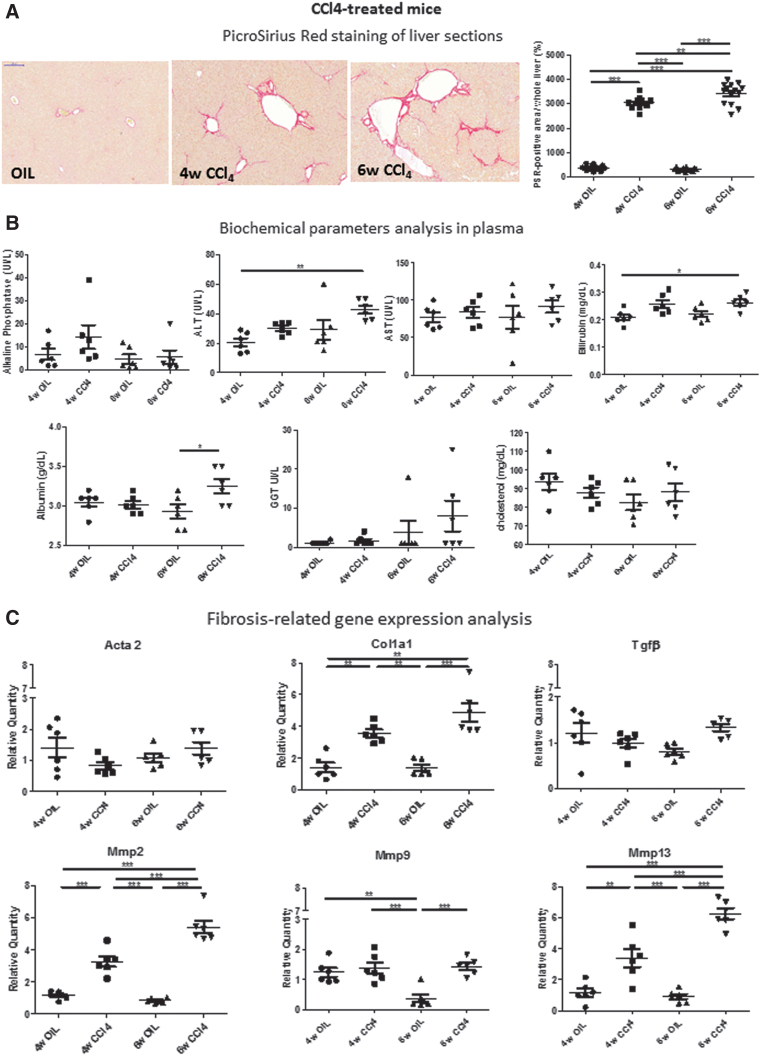

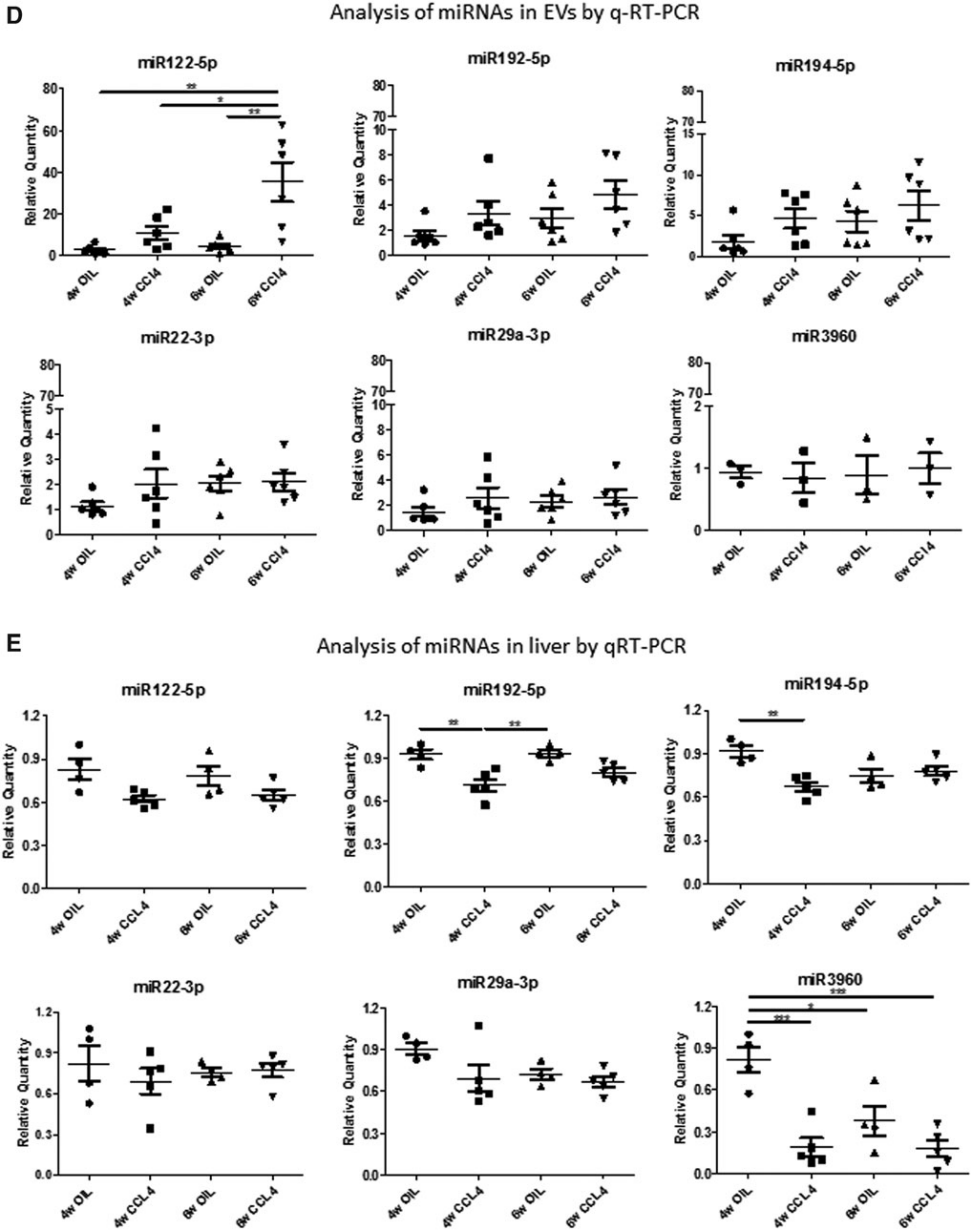
**Analysis of CCl_4_-treated, non-cholestatic mice. (A)** Collagen deposits (*red*) were analyzed by PSR staining in the livers of mice treated with CCl_4_ for 4 and 6 weeks with respect to vehicle-treated mice (OIL) (*n* = 14). **(B)** Biochemical parameters analysis (*n* = 6). **(C)** Fibrosis-related gene expression analysis (*n* = 6). **(D)** miRNAs identified as enriched in circulating by RNA-seq in the BDL model were analyzed by qRT-PCR. Data show mean ± SEM of abundance of each miRNA in circulating EVs (*n* ≥ 4). **(E)** Data show mean ± SEM of abundance of each miRNA in the liver of CCl_4_-treated mice by qRT-PCR (*n* = 3). *p < 0.05, **p < 0.01, ***p < 0.001. OIL, corn oil. Color images are available online.

To assess whether the miRNAs identified as differentially enriched in circulating EVs following severe cholestasis-induced early fibrosis can also be predictive of liver fibrosis in non-cholestatic models, we treated mice with carbon tetrachloride (CCl_4_). As expected, treatment of mice with CCl_4_ for 4 and 6 weeks induced liver fibrosis, as revealed by the PSR staining (Fig. 9A), as well as a statistically significant increase in plasma biochemical parameters (Fig. 9B) and in the expression of fibrosis-related genes (Fig. 9C) but not in liver inflammation-, detoxification/oxidative stress-, and cholangiocyte proliferation-related gene expression, as observed for the severe cholestatic models (Supplementary Fig. S2).

Analysis of EV RNA contents did not show any statistically significant increase in the levels of most selected miRNAs (*i.e.,* miR192-5p, miR194-5p, miR22-3p, miR29a-3p, and miR-3960) following CCl_4_ treatment (Fig. 9D). However, miR122-5p, a marker of hepatocellular injury, significantly increased in the EVs of CCl_4_-treated mice at both 4 and 6 weeks after treatment. Interestingly, liver miR122-5p levels did not change significantly following CCl_4_ treatment compared with oil-treated controls. miR192-5p and miR194-5p expression levels transiently decreased in the liver after 4 weeks of CCl_4_ treatment but reached levels comparable to control mice after 6 weeks of treatment.

## Discussion

Liver fibrosis evolution and resolution are etiology-driven, and general biomarkers may not be sufficient to detect fibrotic development in all liver diseases (38, 39). Our study was designed to provide the basis for translational research on humans by identifying potential biomolecules indicative of cholestasis-induced liver fibrosis in mouse models, and promising as candidate biomarkers for early detection of liver fibrosis in cholestatic patients, for which further studies are warranted. We focused our study on EVs from bloodstream. These vesicles fulfill the criteria of a desirable biomarker (30) since they can carry profibrotic signals or anti-fibrotic molecules depending on the pathophysiological status of their originating organ (8, 28, 41).

EV size and concentration have been found to change in several liver disease settings indicating that EV-based liquid biopsy analysis can be employed for diagnostics, thus warranting further studies on the biological basis or functional relevance (31). For instance, it was shown that, in cirrhotic patients, there was an increase in size, concentration, and protein content of urinary EVs compared with controls (22). Moreover, Wetmore *et al.* reported that the diameter of circulating EV increased in rodents after treatment with D-galactosamine, whereas Cho *et al.* found an increased release of EV after incubation of mice and human hepatocyte cultures with acetaminophen, hence supporting our data (14, 48).

The molecular content of EVs can be useful for monitoring the stage of liver injury and early development of fibrosis since it has been pointed out that every hepatic cell type is an EV producer and/or recipient cell and EVs can carry cues for activating fibroblasts, which can initiate and perpetuate fibrosis (40). Our data show that circulating EVs derived from severe cholestatic mouse models are rich in several RNA species, potentially important as biomarkers. Specifically, miR192-5p, miR194-5p, miR22-3p, and miR29a-3p were consistently highly present in circulating EVs in BDL and DDC-treated mice but not in the mild cholestatic *Mdr2-/-* or non-cholestatic CCl_4_-treated mice.

Notably, the increment of miR29a-3p, which is a major regulator of genes associated with fibrosis, in circulating EVs, reflects the changes observable in the hepatic tissue since miR29a-3p expression augmented in the livers of these mice with time (17). Furthermore, we observed that the abundance of miR29a-3p in EVs correlated with the development of liver fibrosis. Of note, high circulating levels of miR29a-3p were previously shown to be associated with biliary atresia, in particular, in cholestatic pediatric liver disease (21). Moreover, miR194-containing EVs release from hepatocytes is associated to bile acid overload (13).

Considering the link of the miRNAs under study with ex tracellular matrix remodeling (Fig. 4D), we believe that EV-associated miR192-5p, miR194-5p, miR22-3p, and miR29a-3p are promising indicators for severe cholestasis-induced liver fibrosis, and if coupled with miR122-5p, a hepatocellular injury-linked miRNA, these could develop into an optimal instrument for early diagnosis of liver fibrosis (34).

The mRNAs found in cholestatic liver-derived EVs are mainly of hepatic origin, as shown by the presence of miR122-5p, which is abundantly expressed in the liver and massively released upon injury. Moreover, enrichment of hepatic mRNAs (Supplementary Fig. S6 and Supplementary Table S5) playing a clinically significant role in hepatic function, including acute phase response, heme, and iron metabolism was found.

Analysis of the abundance of mRNAs in EVs with time revealed that, following BDL, there was a statistically significantly higher level of *Hp* mRNA. Interestingly, HP is one of the components of FibroTest and ActiTest for the prediction of fibrosis and necroinflammatory activity in patients with chronic liver disease (35). EVs derived from BDL mice were also statistically significantly enriched with *Albumin* mRNA, despite the fact that no statistically significant changes were observed in *Albumin* expression in the liver or in whole serum. This was characteristic of the obstructive cholestasis model.

**FIG. 10.**
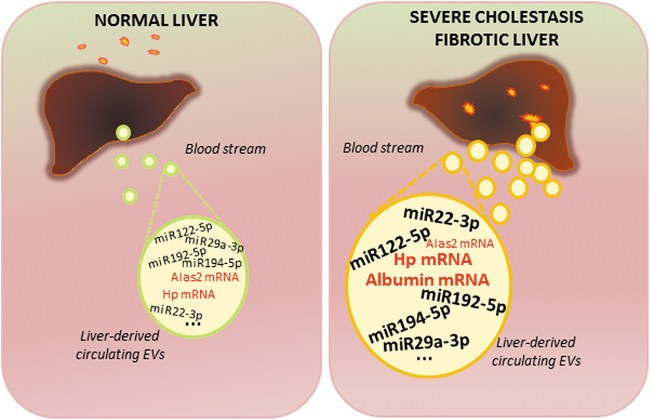
**Schematic representation of biomarkers-containing EVs release from the fibrotic liver.** Under normal conditions, physiological quantities of EVs containing liver-derived biomolecules are released. Upon cholestasis, the damage that occurs in the liver induces massive release of EVs that are enriched with bioactive molecules such as miRNAs and mRNAs, which provide a molecular fingerprint of the underlying pathological events in the liver and can thus be used as biomarkers. Color images are available online.

Thus, the combined use of miRNAs and mRNAs may assist in finding the origin of circulating EVs, in detecting in a noninvasive way any pathological changes occurring in the tissues of origin, and in developing assays such as those based on flow cytometry methodology to allow multiple biomarkers detection in a single setting (Fig. 10) (51). Overall, these findings suggest that, while miR122-5p can be regarded as a generic hepatocellular toxicity marker, circulating EV-contained miR192-5p, miR194-5p, miR22-3p, and miR29a-3p can be specifically predictive of severe cholestasis-induced liver fibrosis.

## Materials and Methods

### Animal models

All experiments were performed in accordance with the Italian legislation on protection of animals (protocol number: CC652.109) and the University of Turin Guidelines. For the BDL procedure, male C57BL/6J mice (in-house breeding) at 8 weeks of age were kept on water and standard laboratory chow *ad libitum*. Mice were anesthetized by intramuscular injection of a cocktail of tiletamine–zolazepam (80 mg/kg) and xylazine (16 mg/kg), and BDL was performed after midline laparotomy. The common bile duct was ligated at two distal points, and the body wall was sutured followed by skin closure with stainless steel wound clips.

Sham operation was performed similarly, except for ligation of the bile duct. All surgical procedures were performed under aseptic conditions. Animals were allowed to recover from anesthesia and surgery under a red warming lamp and were held in pathogen-free cages. The survival rate of mice was monitored and mice were sacrificed at 4, 8, 15, and 21 days after BDL. Friend leukemia virus B (FVB) *Mdr2-/-* mice were purchased from Jackson Laboratories, bred in homozygous condition, and 4 and 6 weeks old males were used for experiments. Age-matched WT FVB mice (in-house breeding, not littermates) were used as controls. Two months old Balb/c male mice (in-house breeding) were fed a 0.1% DDC-supplemented diet for 1 and 2 weeks.

DDC-supplemented diet was produced by adding 0.1% DDC to the 4RF21 diet (Mucedola s.r.l., Italy). Control mice were fed the same chow without DDC. CCl_4_-induced (Sigma–Aldrich, Milano, Italy) hepatic fibrosis was achieved by injecting CCl_4_ resuspended in corn oil biweekly as intraperitoneal injections in a dose of 0.6 μL/g body weight as a 20% solution for 4 or 6 weeks (47). Male WT C57BL/6J mice at 4–6 weeks of age weighing 20–25 g were used for this study. Corn oil (OIL)-treated aged-matched mice were used as controls. During sacrifice, animals were anesthetized, and blood and the liver were taken for analysis.

### Biochemical parameters measurement

Blood was collected into K2 EDTA-coated S-Monovette^®^ by cardiac puncture. Plasma was prepared within 1 h of blood sampling by centrifuging at 1000 *g* for 10 min at 4°C, followed by a second centrifugation of the supernatant at 2000 *g* for 15 min and frozen at −80°C till further processing. Biochemical analyses (AST, ALT, ALP, bilirubin, GGT, total cholesterol, albumin) were performed on BT3500VET (Biotecnica Instruments SpA; Veterinary Science Department, Grugliasco, Turin), whereas high-density lipoprotein was analyzed by standard techniques at the Biochemistry Laboratory (Baldi e Riberi—Città della Salute e delle Scienze, Turin).

### Histology, immunohistochemistry, and image analysis

Five-micrometer sections of formalin-fixed paraffin-embedded liver tissues were stained with hematoxylin and eosin (H/E) for histological examination of main cholestatic features (bile infarct areas, ductular reaction, biliary hyperplasia). For quantification of collagen deposition, PSR staining was performed. Slides were digitized with Panoramic Desk Scanner (3D HISTECH) and subsequently evaluated for collagen deposition, using the ImageJ software, as a percentage of Sirius red-stained area compared with the total section area. To avoid sampling bias, larger portal tracts were excluded from image analysis.

Five-micrometer thick sections were also processed for immunohistochemistry analysis using anti-F4/80 (for macrophages; Serotec) and anti-CD18 (for leukocytes; BMA Biomedicals) antibodies, as previously described (19).

### EV enrichment, characterization, and protein analysis

Circulating EVs were collected from 250 μL of plasma. Briefly, plasma was thawed on ice, and 63 μL of Exoquick™ (System Biosciences) was added to EV preparation according to the manufacturer's instructions. EVs were visualized on the NanoSight LM10 instrument (Particle Characterization Laboratories, Novato). The particle size profile and concentration in plasma samples were evaluated with NTA 3.2 software.

For DLS, isolated EVs were resuspended in phosphate-buffered saline (PBS), and size as well as size distributions of EVs were analyzed (Zetasizer Nano S, Malvern, UK at ECSIN—Laboratorio di ECAMRICERT SRL a Mérieux NutriSciences Company, Padova). All samples were measured at 25°C using a 633 nm laser light set at a scattering angle of 90°, following an equilibration time of 120 s. Analysis was performed using the Malvern Panalytical Zetasizer Ver. 7.13 software.

Proteins from EVs were extracted using RIPA buffer (Sigma) and then quantified using the Bradford method (Bio-Rad), according to the manufacturer's instructions. Ten micrograms of protein was subjected to Western blot analysis for determination of enrichment of exosomal markers (CD9, CD63, CD81, HSP90) in EVs compared with whole plasma, as previously described (Supplementary Fig. S7) (11).

EV internalization in HepG2 cells was also tested. For this purpose, EVs were stained with Vybrant DiO dye (Thermo Fisher Scientific) according to the manufacturer's instructions; excess dye was removed after two steps of washing and ultracentrifugation, as previously described (23). EVs were imaged after labeling using a Leica inverted microscope (DM IL). Moreover, HepG2 cells were incubated with 10^9^ EVs for 24 h, washed, and observed under a Zeiss microscope and Apotome software.

### EV RNA extraction and RNA-seq analysis

The procedure is summarized in Supplementary Figure S2. For total RNA purification, EV pellets were resuspended in 200 μL PBS and 1 mL of QIAzol Lysis Reagent (Qiagen) added. RNA was extracted using miRNeasy Serum/Plasma Kit (Qiagen) in an automated way using the QIAcube instrument (Qiagen), thus preventing operator-depending variability. Total RNA was estimated quantitatively with Qubit RNA HS kit (Thermo Fisher Scientific) and then stored at −80°C till further use.

### EVs-small RNA-seq

Small RNA libraries were prepared using Small RNA Library Preparation Kit NEBNext (New England Biolabs) according to the manufacturer's instructions starting from 40 ng of EV-derived RNA and using 15 PCR cycles. Each library was analyzed with the High Sensitivity DNA chip (Agilent) using Agilent 2100 Bioanalyzer. Pools of 12 libraries were concentrated with AMPure XP magnetic beads (Beckman Coulter) and eluted in 40 μL of nuclease free water.

The eluate from each pool was loaded onto a 6% precast gel (Life Technologies), and a ≈146 bp band was cut and put in 300 μL of nuclease free water overnight in 2 mL low bind Eppendorf tube. Gel debris was removed with QIAquick Gel Extraction Kit (Qiagen), and cDNA was precipitated using 3*M* NaOAc, 100% cold ethanol and glycogen, centrifuged at 20,000 *g* for 20 min at 4°C. After one wash with 70% ethanol, cDNA was resuspended in 10 μL nuclease free water and quantified using the Qubit DNA HS (Life Technologies). Pool was analyzed with the High Sensitivity DNA Chip (Agilent) using Agilent 2100 Bioanalyzer. Two pools (2 × 12 samples purified indexed libraries) were run at the concentration of 1.6 pM on the NextSeq500 sequencer (Illumina) according to the manufacturer's instructions, in 75 nts single-end sequencing mode.

### EVs-whole transcriptome sequencing

To detect coding and long non-coding RNAs present in EVs, the TruSeq stranded mRNA library preparation kit (Illumina) was used, following the manufacturer's instructions but skipping the depletion of ribosomal RNAs and purification of the polyA+ fraction phases. Briefly, 40 ng of total RNA extracted from EVs was fragmented for 8 min at 94°C. First-strand and second-strand cDNA were synthetized, a single A nucleotide was added to the 3′ ends of the blunt fragments, and multiple indexing adapters were ligated at the end of the double-strand cDNA.

A 15 cycles PCR was performed to selectively enrich those DNA fragments with adapter molecules on both ends. Each library was analyzed with the DNA 1000 chip (Agilent) using Agilent 2100 Bioanalyzer and quantified using the Qubit DNA HS Kit (Life Technologies). A pool of 12 libraries (pooled at equimolar concentration) was generated, quantified with Qubit DNA HS kit, and run at the concentration of 1.6 pM on the NextSeq500 (Illumina) sequencer in 75 nts paired-end sequencing mode following the manufacturer's instructions.

### Bioinformatics analysis

All analyses were performed using the tools embedded in docker4seq package (https://pubmed.ncbi.nlm.nih.gov/29069297), to guarantee bioinformatically reproducible results (https://pubmed.ncbi.nlm.nih.gov/30367595/). In brief, miRNAseq quantification was performed using the workflow described by Cordero and coworkers (16), using miRBase 21.

Whole transcriptome RNA-seq quantification was performed using STAR/RSEM (18, 27). Reads were mapped against human genome (hg38) using ENSEMBL GTF (version 99) annotation. Differential expression analysis was performed using DESeq2 Bioconductor package (4). RNAs with a |Log_2_FC| ≥ 1 and an adjusted *p* value ≤0.1 were considered differentially expressed. PCA, Venn diagrams, and unsupervised hierarchical clustering were used to inspect and compare the expression data. Differentially expressed miRNAs found in the BDL mouse model were assessed for conservation in humans, on the basis of their perfect sequence identity among human and mouse in mirbase database (https://www.mirbase.org). Electronic laboratory notebook was not used.

### RNA extraction and qRT-PCR analysis

Total RNA was isolated from the liver tissue samples using PureLink RNA Mini Kit (Thermo Fisher Scientific), as previously described (3). For quantitative gene expression analysis, we used the Roche UPL library and primers (Sigma) according to Supplementary Table S1 and run on a Quantstudio Flex 6 Real-Time PCR Systems. TaqMan custom gene expression assay panels were also used for some experiments (Supplementary Table S2). Data were analyzed using the ddCt method. Transcript abundance, normalized to 18 s mRNA expression, was expressed as a fold change over a calibrator sample.

For miRNA analysis, RNA was extracted from EVs or from the liver as described above, and analyzed using miRCURY locked nucleic acid (LNA) miRNA PCR assays (Qiagen) or panels (Supplementary Table S2) according to the manufacturer's instructions. Internal housekeeping miRNAs were selected from RNA-seq data. As comparable results were obtained by using two chosen unmodulated miRNAs, miR320-3p and Let7b, all successive normalizations were performed using Let7b.

### Statistical analysis

One-way analysis of variance (ANOVA) followed by Bonferroni's *post hoc* test was performed to compare between multiple experimental groups using GraphPad Prism5 software. *p < 0.05, **p < 0.01, and ***p < 0.001 were considered statistically significant.

## Supplementary Material

Supplemental data

Supplemental data

Supplemental data

Supplemental data

Supplemental data

Supplemental data

Supplemental data

Supplemental data

Supplemental data

Supplemental data

Supplemental data

Supplemental data

## Data Availability

Data are available at the GEO repository (https://www.ncbi.nlm.gov/geo/query/acc.cgi?acc=GSE166754).

## Abbreviations Used

ALP: alkaline phosphatase
ALT: alanine aminotransferase
ANOVA: analysis of variance
AST: aspartate aminotransferase
BDL: bile duct ligation
CCl_4_: carbon tetrachloride
Col1a1: collagen 1A1
Cxcl2: C-X-C motif chemokine ligand 2
DAPI: 4′,6-diamidino-2-phenylindole
DDC: 3,5-diethoxycarboncyl-1,4-dihydrocollidine
DiO: DiOC18(3) (3,3′-dioctadecyloxacarbocyanine perchlorate)
DLS: dynamic light scattering
EVs: extracellular vesicles
FVB: Friend leukemia virus B
GGT: gamma glutamyl transferase
Gsta2: glutathione S-transferase alpha 2
H/E: hematoxylin and eosin
Hp: haptoglobin
Il-1β: interleukin-1β
LNA: locked nucleic acid
MDR: multidrug resistance P-glycoprotein
miRNA: microRNA
MMP: metalloproteinase
NT: nontreated mice
NTA: nanoparticle tracking analysis
PBS: phosphate-buffered saline
PCA: principal component analysis
PCR: polymerase chain reaction
PSC: primary sclerosing cholangitis
PSR: PicroSirius red
qRT-PCR: quantitative real-time PCR
RNA-seq: RNA sequencing
SEM: standard error of the mean
TfR1: transferrin receptor 1
TGF: transforming growth factor
Tnfα: tumor necrosis factor α
WT: wild type
