# Supplementary material for: Circulating Extracellular Vesicles Contain Liver-Derived RNA Species as Indicators of Severe Cholestasis-Induced Early Liver Fibrosis in Mice
Source: Antioxid Redox Signal. 2022 Mar 17;36(7-9):480–504. doi: 10.1089/ars.2021.0023 (PMC8978575; doi:10.1089/ars.2021.0023)
Supplement: Supplemental data [file Suppl_FigS1.docx]

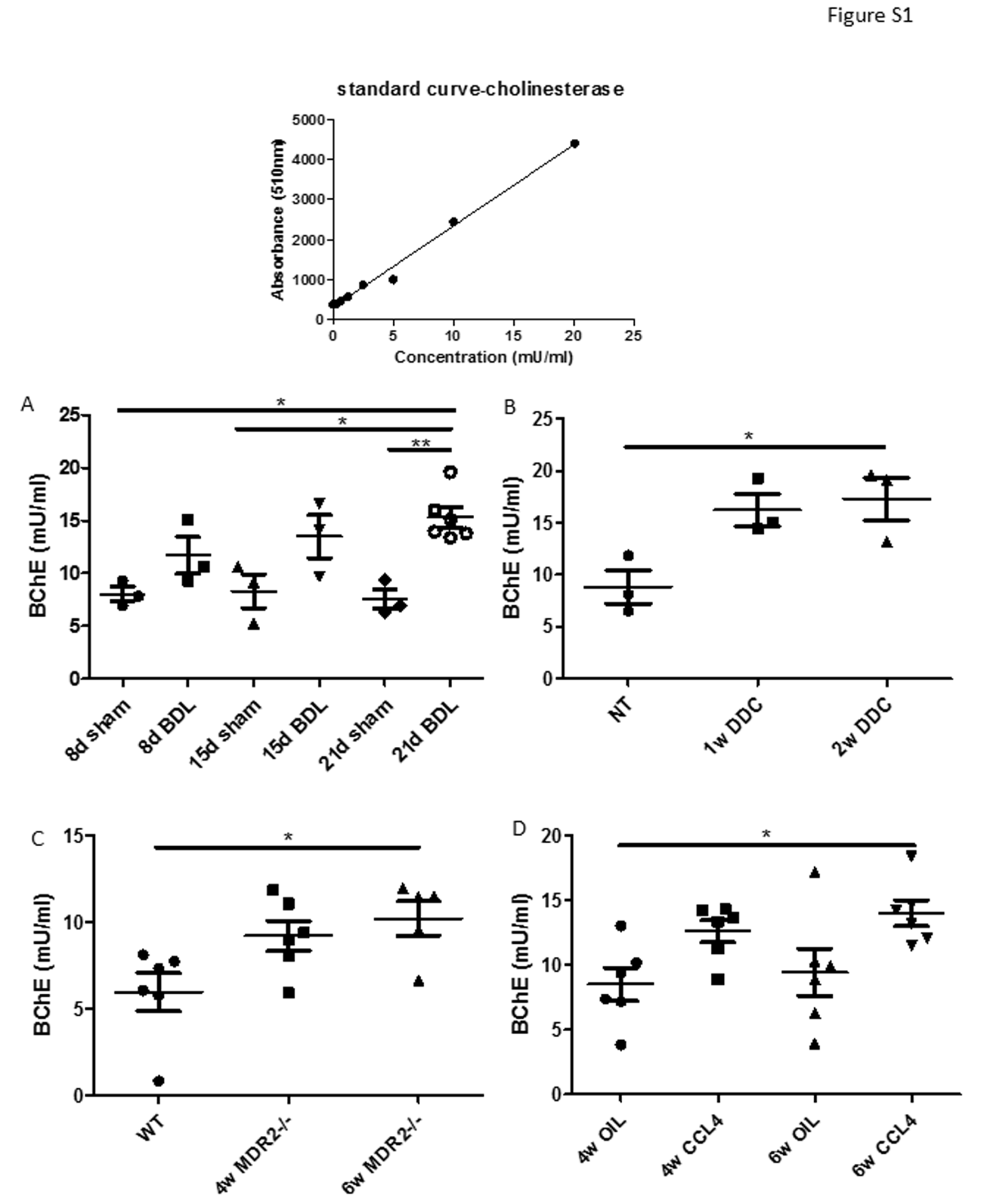


**Fig.S1: Serum cholinesterase activity in the four mouse models employed in this study**. Butyrylcholinesterase (BChE) activity was measured in serum of (A) BDL, (B) DDC-treated, (C) *Mdr2-/-* and (D) CCl_4_-treated mice. **p*<0.05, ***p*<0.01
