# Supplementary material for: Circulating Extracellular Vesicles Contain Liver-Derived RNA Species as Indicators of Severe Cholestasis-Induced Early Liver Fibrosis in Mice
Source: Antioxid Redox Signal. 2022 Mar 17;36(7-9):480–504. doi: 10.1089/ars.2021.0023 (PMC8978575; doi:10.1089/ars.2021.0023)

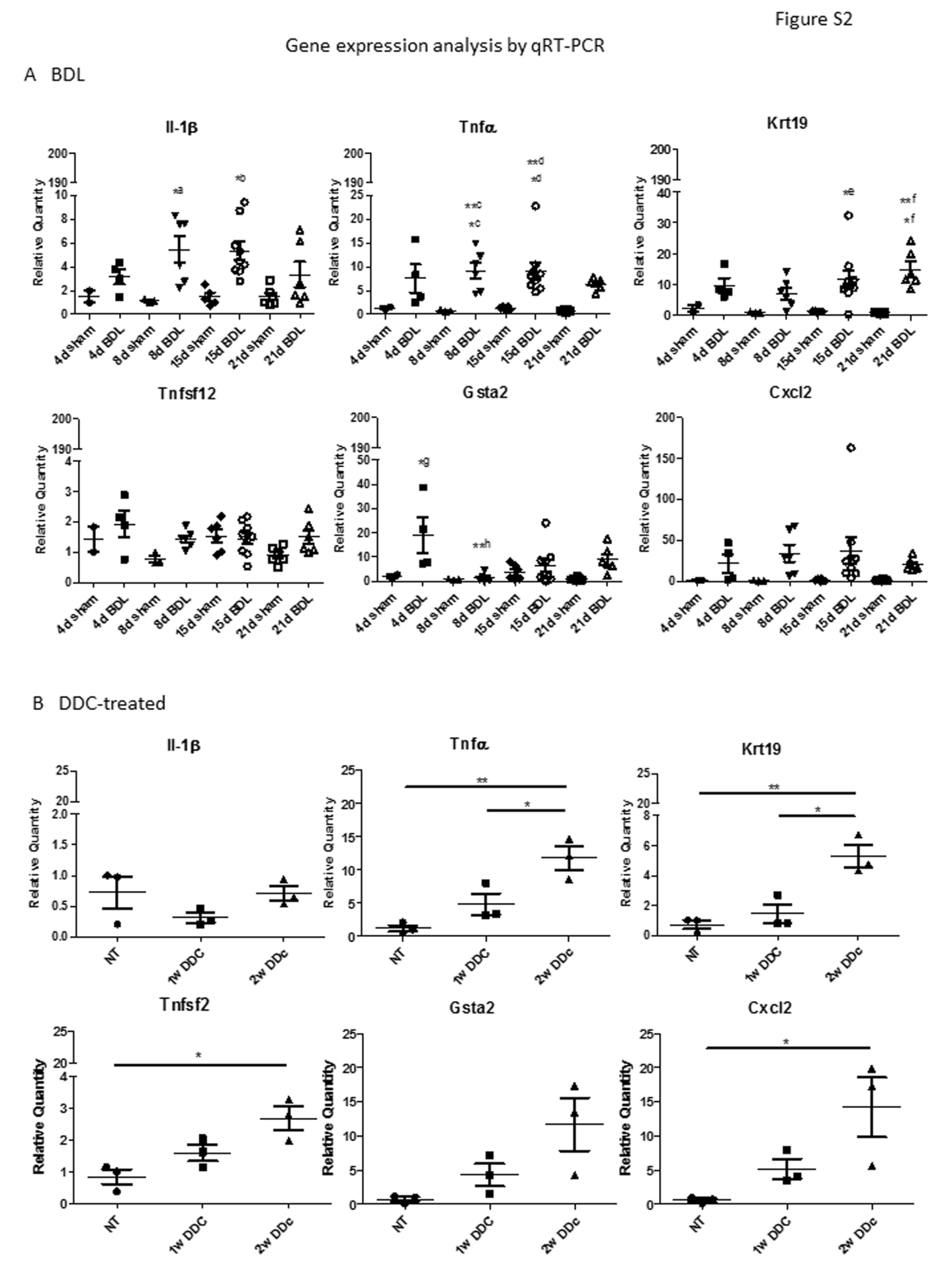


**Fig.S2: Gene expression analysis by qRT-PCR on liver tissue.** Interleukin-1β (*Il-1β*),

Tumor necrosis factor α (*Tnfα*), Keratin 19 (*Krt19*); Tumor necrosis factor ligand superfamily member 12 (*Tnfsf12*), Glutathione S-Transferase Alpha 2 (*Gsta2*); C-X-C Motif Chemokine Ligand 2 (*Cxcl2*) were analysed by qRT-PCR in (A) BDL, (B) DDC-treated, (C) *Mdr2-/-* and (D) CCl_4_-treated mice. *a: 8d BDL *vs.* 21d sham, *b: 15d BDL *vs.* 15d sham and 21d sham, *c: 8d BDL *vs.* 15d sham, **c: 8d BDL *vs.* 21d sham, *e: 15d BDL *vs.* 15d sham and 21d sham, *f: 21d BDL *vs.* 8d sham, **f: 21d BDL *vs.* 15d sham and 21d sham, *g: 4d BDL *vs.* 8d sham, 15d sham and 21d sham, **h: 8d BDL *vs.* 4d BDL; **p*<0.05, ***p*<0.01, ****p*<0.001.


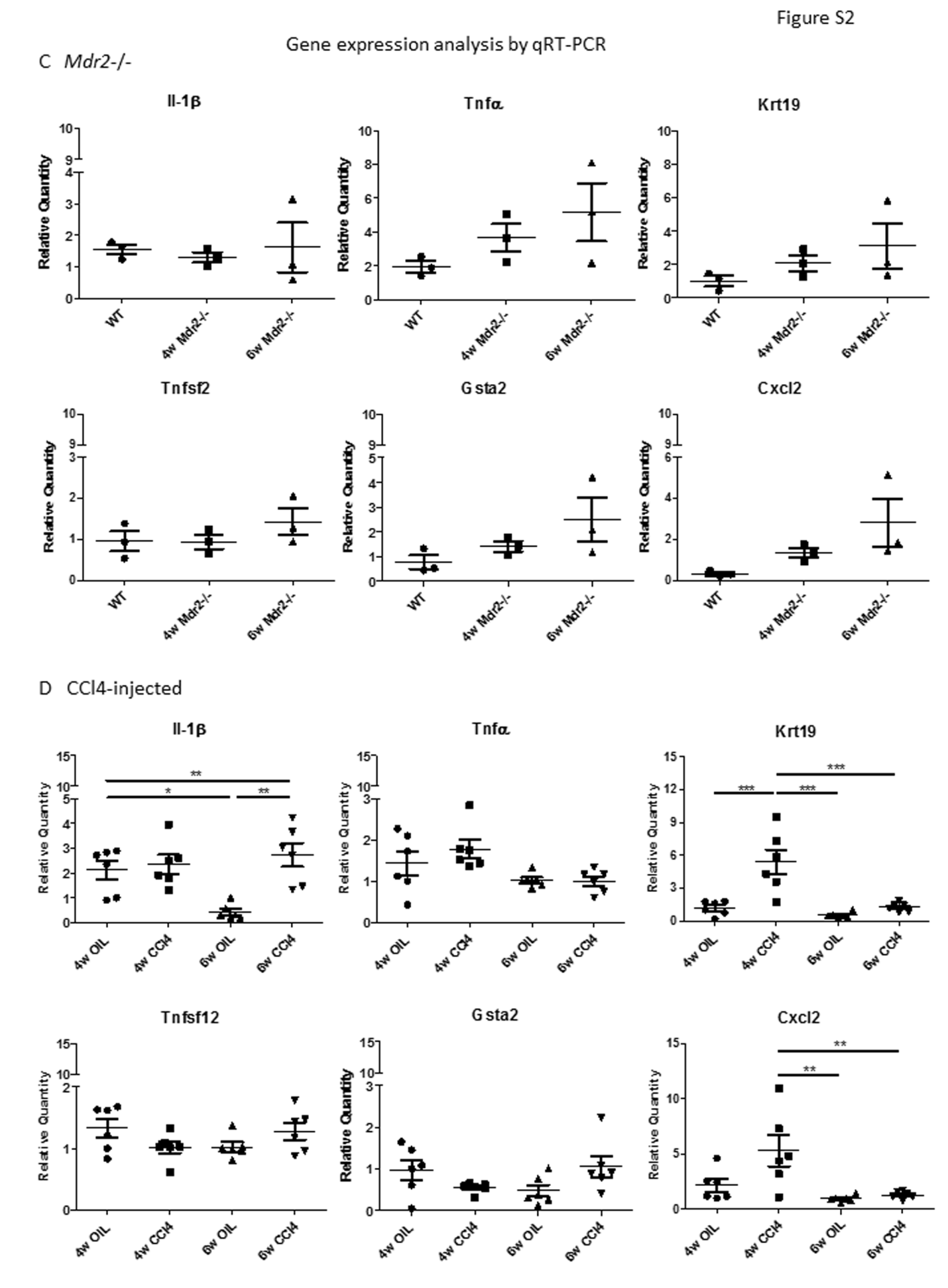

Supplement: Supplemental data [file Suppl_FigS2.docx]
