# Supplementary figures and images for: Circulating Extracellular Vesicles Contain Liver-Derived RNA Species as Indicators of Severe Cholestasis-Induced Early Liver Fibrosis in Mice
Source: Antioxid Redox Signal. 2022 Mar 17;36(7-9):480–504. doi: 10.1089/ars.2021.0023 (PMC8978575; doi:10.1089/ars.2021.0023)

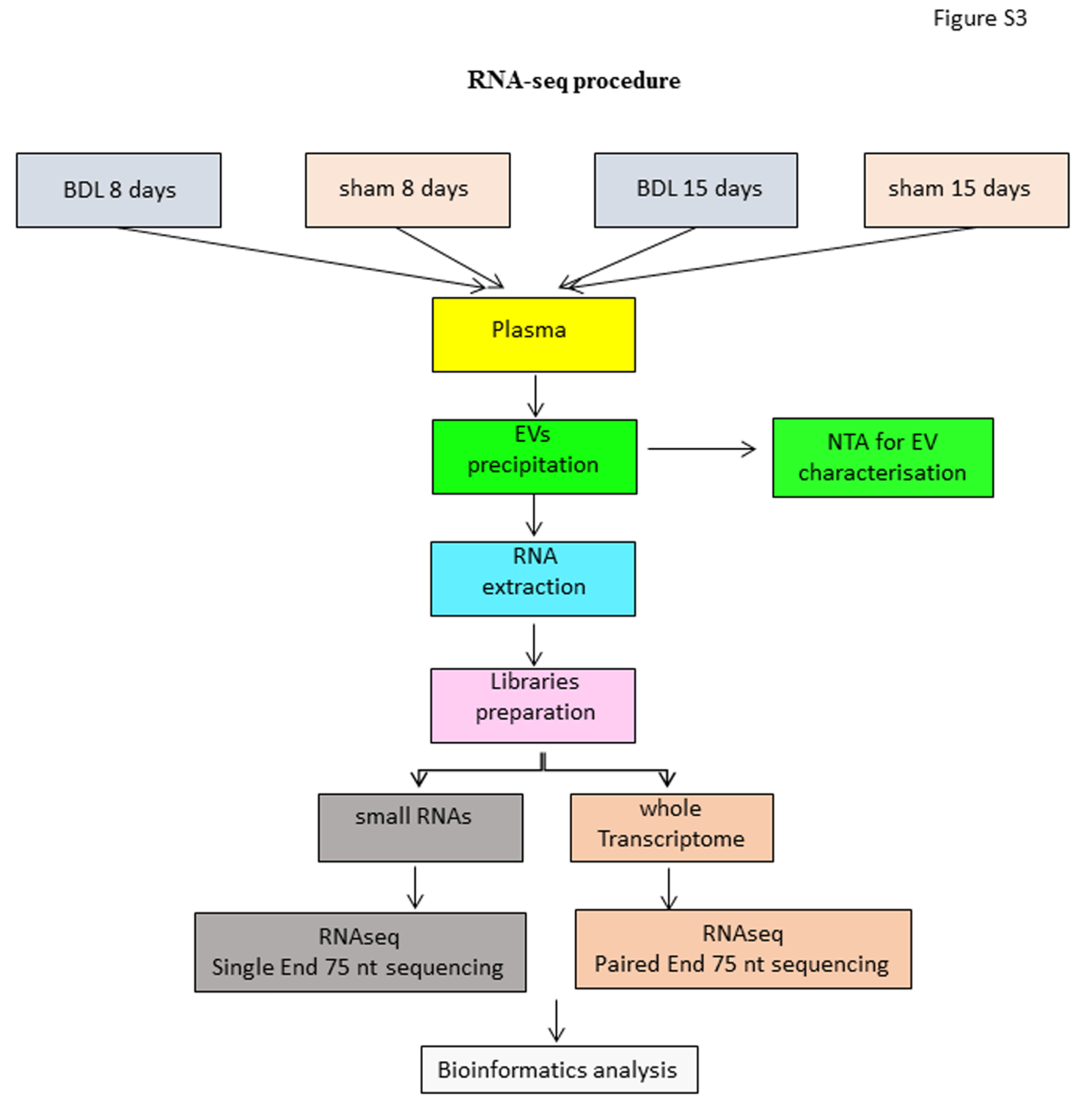


**Fig.S3: The RNA-seq procedure used in this study.**

Supplement: Supplemental data [file Suppl_FigS3.docx]

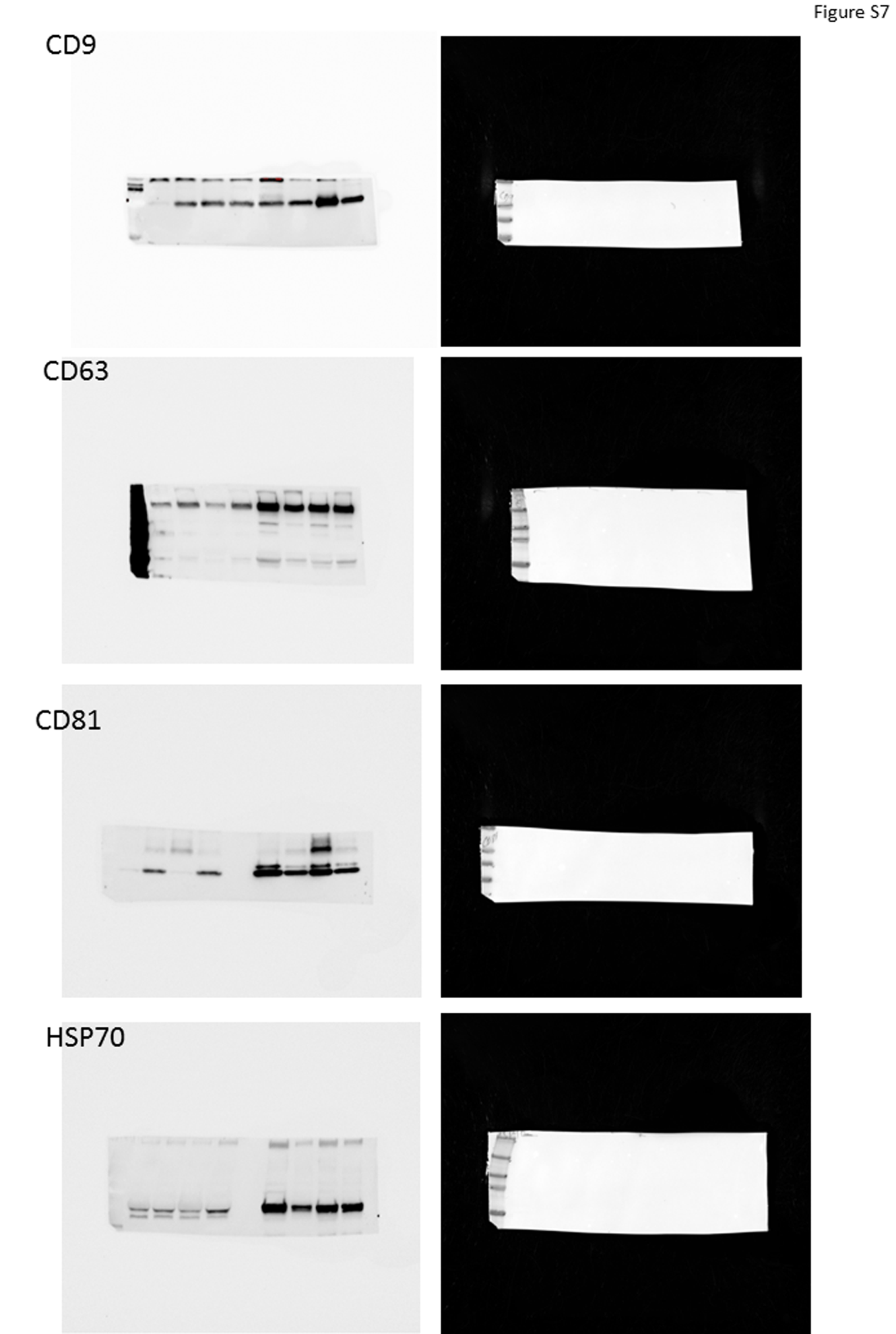


**Fig.S7: Uncropped western blot images from Figure 3A.**

Supplement: Supplemental data [file Suppl_FigS7.docx]
