# Supplementary material for: Circulating Extracellular Vesicles Contain Liver-Derived RNA Species as Indicators of Severe Cholestasis-Induced Early Liver Fibrosis in Mice
Source: Antioxid Redox Signal. 2022 Mar 17;36(7-9):480–504. doi: 10.1089/ars.2021.0023 (PMC8978575; doi:10.1089/ars.2021.0023)
Supplement: Supplemental data [file Suppl_FigS4.docx]

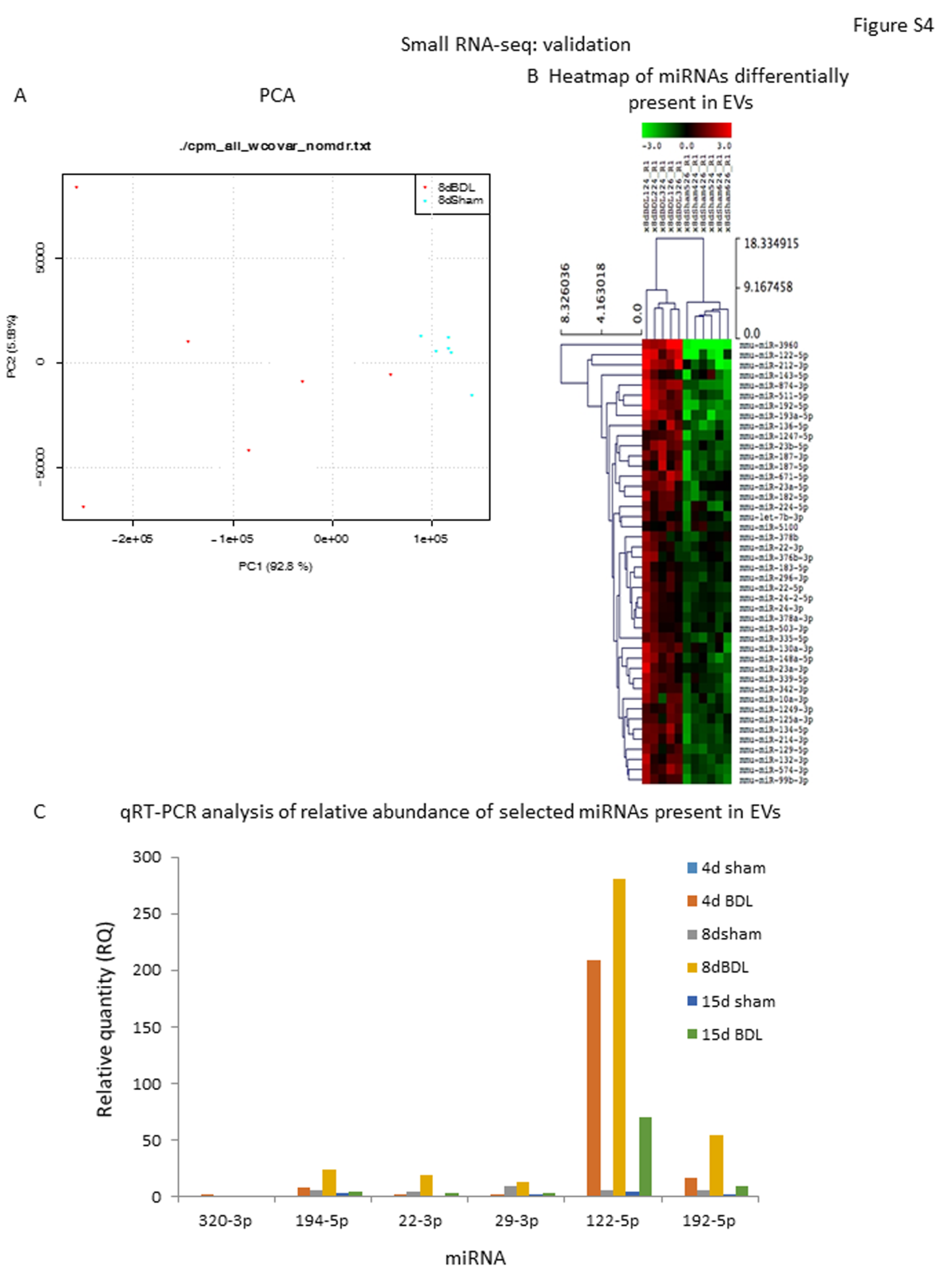


**Fig.S4: Small RNA-seq for validation of results.** A. PCA analysis. B. Heatmap of miRNAs enriched in circulating EVs after 8d BDL with respect to sham controls. C. Analysis of comparative abundance of selected miRNAs by qRT-PCR.
