# Supplementary material for: Circulating Extracellular Vesicles Contain Liver-Derived RNA Species as Indicators of Severe Cholestasis-Induced Early Liver Fibrosis in Mice
Source: Antioxid Redox Signal. 2022 Mar 17;36(7-9):480–504. doi: 10.1089/ars.2021.0023 (PMC8978575; doi:10.1089/ars.2021.0023)
Supplement: Supplemental data [file Suppl_FigS5.docx]

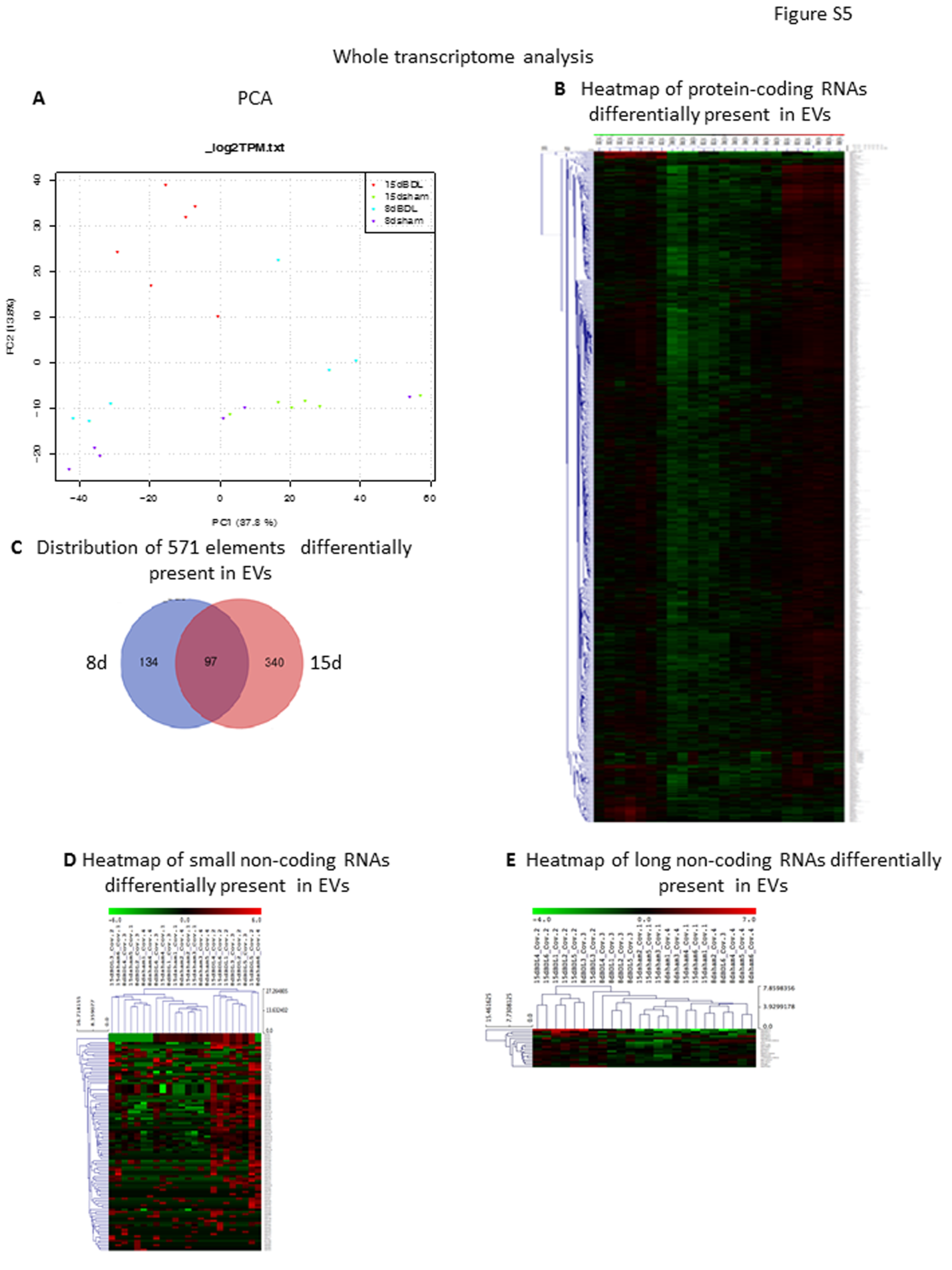


**Fig.S5: Whole transcriptome analysis.** A. PCA analysis. B. Heatmap of all mRNAs differentially present in circulating EVs between BDL and sham-operated mice. C. Distribution of the 571 elements identified in this study and differentially present in the EVs. D. Heatmap of small non-coding RNAs differentially present in circulating EVs. E. Heatmap of long non-coding RNAs differentially present in circulating EVs.
