# Supplementary material for: Circulating Extracellular Vesicles Contain Liver-Derived RNA Species as Indicators of Severe Cholestasis-Induced Early Liver Fibrosis in Mice
Source: Antioxid Redox Signal. 2022 Mar 17;36(7-9):480–504. doi: 10.1089/ars.2021.0023 (PMC8978575; doi:10.1089/ars.2021.0023)
Supplement: Supplemental data [file Suppl_TableS4.docx]

| **RNA type** | **number of transcripts with reads > 0 in at least one sample** | **number of transcripts with reads > 5 in at least one sample** |
| --- | --- | --- |
| proteins | 18710 | 10690 |
| lincRNA | 1911 | 76 |
| snoRNA | 138 | 40 |
| snRNA | 96 | 37 |
| antisense | 1144 | 31 |
| miRNA | 25 | 11 |
| scaRNA | 18 | 9 |
| misc_rna | 48 | 8 |
| sense_intronic | 148 | 3 |
| sense_overlapping | 14 | 1 |
| macro_lincRNA | 0 | 0 |
| non_coding | 0 | 0 |
| scRNA | 0 | 0 |
| sRNA | 0 | 0 |
| vaultRNA | 0 | 0 |

Table S4: RNA species identified with whole transcriptome analysis.
