# Supplementary material for: Circulating Extracellular Vesicles Contain Liver-Derived RNA Species as Indicators of Severe Cholestasis-Induced Early Liver Fibrosis in Mice
Source: Antioxid Redox Signal. 2022 Mar 17;36(7-9):480–504. doi: 10.1089/ars.2021.0023 (PMC8978575; doi:10.1089/ars.2021.0023)
Supplement: Supplemental data [file Suppl_FigS6.docx]

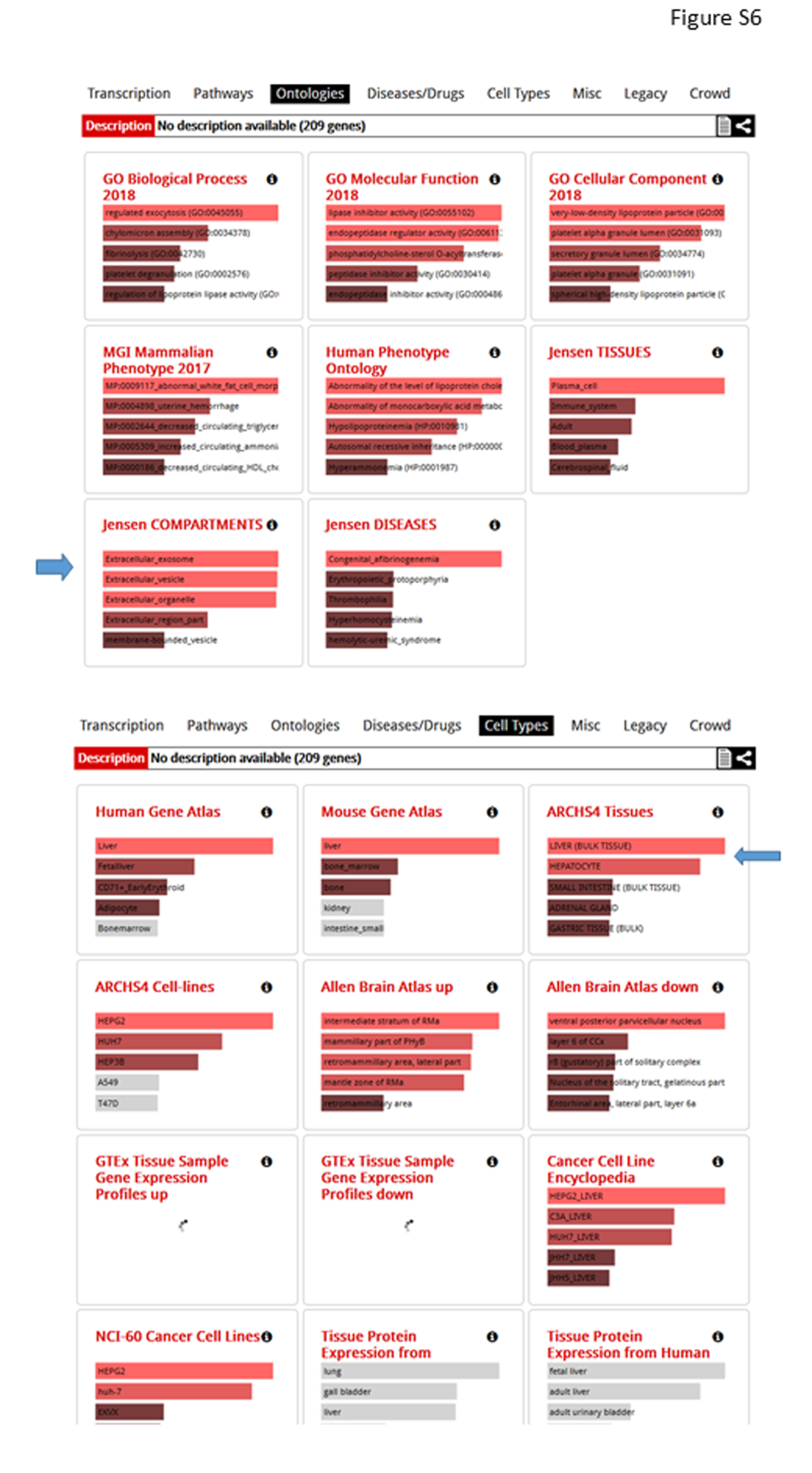


**Fig.S6: EnrichR analysis of mRNAs enriched in EVs following BDL.** Arrows point out to the main sources of mRNAs.
