# Supplementary material for: Circulating Extracellular Vesicles Contain Liver-Derived RNA Species as Indicators of Severe Cholestasis-Induced Early Liver Fibrosis in Mice
Source: Antioxid Redox Signal. 2022 Mar 17;36(7-9):480–504. doi: 10.1089/ars.2021.0023 (PMC8978575; doi:10.1089/ars.2021.0023)
Supplement: Supplemental data [file Suppl_TableS1.docx]

Table S1

| **Gene** | **Primers 5’-3’** | **UPL probe (Roche)** |
| --- | --- | --- |
| Col1A1 | Catgttcagctttgtggacct  gcagctgacttcagggatgt | #15 |
| αSMA | Taacccttcagcgttcagc  acatagctggagcagcgtct | #20 |
| TGFβ | Tggagcaacatgtggaactc  gtcagcagccggttacca | #72 |
| MMP13 | Cagtctccgaggagaaactatgat  ggactttgtcaaaaagagctcag | #62 |
| MMP9 | Agacgacatagacggcatcc  tcggctgtggttcagttgt | #19 |
| MMP2 | gtgggacaagaaccagatcac  gcatcatccacggtttcag | #85 |
| TfR1 | Catgagggaaatcaatgatcg  ctaaagctgagagagtgtgagagc | #45 |
| Albumin | Agtgttgtgcagaggctgac  ttctccttcacaccatcaagc | #27 |
| Hp | Ggcaagagaggtccacgat  ccacagcaaaaagctgacc | #15 |
| Alas2 | Ctcaccgtctttggttcgtc  ggacaggaccgtagcaacat | #74 |
