# Supplementary material for: Circulating Extracellular Vesicles Contain Liver-Derived RNA Species as Indicators of Severe Cholestasis-Induced Early Liver Fibrosis in Mice
Source: Antioxid Redox Signal. 2022 Mar 17;36(7-9):480–504. doi: 10.1089/ars.2021.0023 (PMC8978575; doi:10.1089/ars.2021.0023)
Supplement: Supplemental data [file Suppl_TableS2.docx]

| **Gene symbol** | **Taqman Assay ID** |
| --- | --- |
| 18s | Mm03928990_g1 |
| Col1a1 | Mm00801666_g1 |
| Acta2 | Mm01204962_gH |
| Tgfb1 | Mm01178820_m1 |
| Mmp13 | Mm00439491_m1 |
| Mmp9 | Mm00442991_m1 |
| Mmp2 | Mm00439498_m1 |
| Il1b | Mm00434228_m1 |
| Tnf | Mm00443258_m1 |
| Krt19 | Mm00492980_m1 |
| Tnfsf12 | Mm02583406_s1 |
| Gsta2 | Mm00833353_mH |
| Cxcl2 | Mm00436450_m1 |

Table S2: TaqMan Gene Expression Assays (ThermoFisher Scientific)

miRCURY LNA miRNA custom PCR panel (Qiagen)

| UniSp6 miRCURY LNA miRNA PCR Assay |
| --- |
| hsa-let-7b-5p |
| hsa-miR-192-5p |
| hsa-miR-122-5p |
| hsa-miR-194-5p |
| hsa-miR-29a-3p |
| hsa-miR-22-3p |
| hsa-miR-3960 |
